# Supplementary material for: Development and qualification of quantitative assays for detecting process-related nucleic acid impurities in recombinant AAV preparations
Source: Mol Ther Adv. 2026 Sep 30;34(4):201854. doi: 10.1016/j.omta.2026.201854 (PMC13720811; doi:10.1016/j.omta.2026.201854)
Supplement: Supplementary file 1 [file mmc1.pdf]

OMTA, Volume *34*

Supplemental information

Development and qualification of quantitative assays for detecting process-related nucleic acid impurities in recombinant AAV preparations

Mojca Janc and David Dobnik

Table S1: Digital PCR primers and probes developed and used in this study. Forward and reverse primers were used at the final concentration of 900 nmol/L and probes at 250 nmol/L.

DNA impurity target	Assay number	Assay coordinates on gBlock (bp)	Label	DNA Sequence of oligonucleotide (5' to 3')
**Amp**	1	20–82	Amp_res1 FP	GTGTCGCCCTTATTCCCTTTT
			Amp_res1 RP	GCGTTTCTGGGTGAGCAAA
			Amp_res1 Probe	FAM-TGCGGCATT/ZEN/TTGCCTTCC -BkFQ
	2	219–280	Amp_res2 FP	GCTATGTGGCGCGGTATTATC
			Amp_res2 RP	GTATGCGGCGACCGAGTT
			Amp_res2 Probe	FAM-CGTATTGAC/ZEN/GCCGGGCAAGAG -BkFQ
	3	676–745	Amp_res3 FP	GGCTGGTTTATTGCTGATAAATCT
			Amp_res3 RP	GCCCCAGTGCTGCAATG
			Amp_res3 Probe	HEX-CCGGTGAGC/ZEN/GTGGGTCTCGC -BkFQ
**Kan**	1	75–150	Kan_res1 FP	AAAGCCGTTTCTGTAATGAAGGA
			Kan_res1 RP	GCAGACCGATACCAGGATCTTG
			Kan_res1 Probe	FAM-AAAACTCAC/ZEN/CGAGGCAGTTCCATAGGATG-BkFQ
	2	245–309	Kan_res2 FP	TCCGGTGAGAATGGCAAAA
			Kan_res2 RP	CGTAATGGCTGGCCTGTTG
			Kan_res2 Probe	FAM-CTTATGCAT/ZEN/TTCTTTCCAGACTTG-BkFQ
	3	672–743	Kan_res3 FP	CATCGGGCTTCCCATACAAT
			Kan_res3 RP	GGTATAAATGGGCTCGCGATAA
			Kan_res3 Probe	HEX-TAGATTGTC/ZEN/GCACCTGATTGCCCGA-BkFQ
**SV40T**	1	119–191	LT-ant 1 FP	GCCTGTTTCATGCCCTGAGT
			LT-ant 1 RP	CAGCCAGGAAAATGCTGATAAA
			LT-ant 1 Probe	FAM-TCCATGTTC/ZEN/TTCTCCCCACCATCTTCA-BkFQ
	2	1812–1895	LT-ant 2 FP	TCCAATTATGTCACACCACAGAAGT
			LT-ant 2 RP	GCTACTGCTTCGATTGCTTTAGAA
			LT-ant 2 Probe	FAM-AGGTTCCTT/ZEN/CACAAAGATCAAGTCCAAACCA-BkFQ
	3	2190–2274	LT-ant 3 FP	AAATATGCCTTTCTCATCAGAGGAA
			LT-ant 3 RP	GAGAGGAATCTTTGCAGCTAATGG
			LT-ant 3 Probe	HEX-TCCCCCAGG/ZEN/CACTCCTTTCAAGACCTA-BkFQ
**E1A**	1	45–113	E1a 1 FP	GGCCGCCAGTCTTTTGG
			E1a 1 RP	AAATGGCTAGGAGGTGGAAGATT
			E1a 1 Probe	FAM-CCAGCTGAT/ZEN/CGAAGAGGTACTGGCTGA-BkFQ
	2	874–940	E1a 2 FP	AACCAGTTGCCGTGAGAGTT
			E1a 2 RP	TCGTTAAGCAAGTCCTCGATACAT
			E1a 2 Probe	HEX-TGGGCGTCG/ZEN/CCAGGCTGTG-BkFQ

Table S2: Full-length DNA sequences of plasmid derived gBlocks with marked dPCR assay coordinates. All assay coordinates are shown in bold; additionally, assay 2 is double-underlined and assay 3 is dashed-underlined.

gBlock name	DNA impurity target	gBlock length (bp)	Assay	Assay start and end position	gBlock sequence (5' to 3')
**Amp**	Ampicillin resistance gene	861	1<br>2<br>3	**20–82**<br>**219–280**<br>**676–745**	1 ATGAGTATTCAACATTTCC**GTGTCGCCCTTATTCCCTTTTTTGCGGCATT**<br>51 **TTGCCTTCCTGTTTTTGCTCACCCAGAAACGC**TGGTGAAAGTAAAAGATG<br>101 CTGAAGATCAGTTGGGTGCACGAGTGGGTTACATCGAACTGGATCTCAAC<br>151 AGCGGTAAGATCCTTGAGAGTTTTCGCCCCGAAGAACGTTTTCCAATGAT<br>201 GAGCACTTTTAAAGTTCT**GCTATGTGGCGCGGTATTATCCCGTATTGACG**<br>251 **CCGGGCAAGAGCAACTCGGTCGCCGCATAC**ACTATTCTCAGAATGACTTG<br>301 GTTGAGTACTCACCAGTCACAGAAAAGCATCTTACGGATGGCATGACAGT<br>351 AAGAGAATTATGCAGTGCTGCCATAACCATGAGTGATAACACTGCGGCCA<br>401 ACTTACTTCTGACAACGATCGGAGGACCGAAGGAGCTAACCGCTTTTTTG<br>451 CACAACATGGGGGATCATGTAACTCGCCTTGATCGTTGGGAACCGGAGCT<br>501 GAATGAAGCCATACCAAACGACGAGCGTGACACCACGATGCCTGTAGCAA<br>551 TGGCAACAACGTTGCGCAAACTATTAACTGGCGAACTACTTACTCTAGCT<br>601 TCCCGGCAACAATTAATAGACTGGATGGAGGCGGATAAAGTTGCAGGACC<br>651 ACTTCTGCGCTCGGCCCTTCCGGCT**GGCTGGTTTATTGCTGATAAATCTG**<br>701**GAGCCGGTGAGCGTGGGTCTCGCGGTATCATTGCAGCACTGGGGC**CAGAT<br>751 GGTAAGCCCTCCCGTATCGTAGTTATCTACACGACGGGGAGTCAGGCAAC<br>801 TATGGATGAACGAAATAGACAGATCGCTGAGATAGGTGCCTCACTGATTA<br>851 AGCATTGGTAA
**Kan**	Kanamycin resistance gene	816	1<br>2<br>3	**75–150**<br>**245–309**<br>**672–743**	1 TTAGAAAAACTCATCGAGCATCAAATGAAACTGCAATTTATTCATATCAG<br>51 GATTATCAATACCATATTTTTGAA**AAAGCCGTTTCTGTAATGAAGGAGAA**<br>101 **AACTCACCGAGGCAGTTCCATAGGATGGCAAGATCCTGGTATCGGTCTGC**<br>151 GATTCCGACTCGTCCAACATCAATACAACCTATTAATTTCCCCTCGTCAA<br>201 AAATAAGGTTATCAAGTGAGAAATCACCATGAGTGACGACTGAA**TCCGGT**<br>251 **GAGAATGGCAAAAGTTTATGCATTTCTTTCCAGACTTGTTCAACAGGCCA**<br>301 **GCCATTACG**CTCGTCATCAAAATCACTCGCATCAACCAAACCGTTATTCA<br>351 TTCGTGATTGCGCCTGAGCGAGACGAAATACGCGATCGCTGTTAAAAGGA<br>401 CAATTACAAACAGGAATCGAATGCAACCGGCGCAGGAACACTGCCAGCGC<br>451 ATCAACAATATTTTCACCTGAATCAGGATATTCTTCTAATACCTGGAATG<br>501 CTGTTTTCCCAGGGATCGCAGTGGTGAGTAACCATGCATCATCAGGAGTA<br>551 CGGATAAAATGCTTGATGGTCGGAAGAGGCATAAATTCCGTCAGCCAGTT<br>601 TAGTCTGACCATCTCATCTGTAACATCATTGGCAACGCTACCTTTGCCAT<br>651 GTTTCAGAAACAACTCTGGCG**CATCGGGCTTCCCATACAATCGATAGATT**<br>701 **GTCGCACCTGATTGCCCGACATTATCGCGAGCCCATTTATACC**CATATAA<br>751 ATCAGCATCCATGTTGGAATTTAATCGCGGCCTAGAGCAAGACGTTTCCC<br>801 GTTGAATATGGCTCAT

Table S3: Full-length DNA sequences of gBlocks derived from host cell DNA impurities with marked dPCR assay coordinates. All assay coordinates are shown in bold; additionally, assay 2 is double-underlined and assay 3 is dashed-underlined.

gBlock name	DNA impurity target	gBlock length (bp)	Assay	Assay start and end position	gBlock sequence (5' to 3')
**mSV40T**	SV40 large T antigen	2293	1<br>2<br>3	**119–191**<br>**1812–1895**<br>**2190–2274**	1 TTATGTTTCAGGTTCAGAAGCAAGTAAAACCTCTACAAATGTGGTATGGC<br>51 TGATTATGATCATGAACAGACTGTGAGGACTGAGGGGCCTGAAATGAGCC<br>101 TTGGGACTGTGAATCAAT**GCCTGTTTCATGCCCTGAGTCTTCCATGTTCT**<br>151 **TCTCCCCACCATCTTCATTTTTATCAGCATTTTCCTGGCTG**TCTTCATCA<br>201 TCATCATCACTGTTTCTTAGCCAATCTAAAACTCCAATTCCCATAGCCAC<br>251 ATTAAACTTCATTTTTTGATACACTGACAAACTAAACTCTTTGTCCAATC<br>301 TCTCTTTCCACTCCACAATTCTGCTCTGAATACTTTGAGCAAACTCAGCC<br>351 ACAGGTCTGTACCAAATTAACATAAGAAGCAAAGCAATGCCACTTTGAAT<br>401 AACAAAAACTCACTGCGTTCCAGGCAATGCTTTAAATAATCTTTGGGCCT<br>451 AAACAAATCTGGCCTGCAGTCACACTGTACTCATTCATGGTGACTATTCC<br>501 AGGGGGAAATATTTGAGTTACCTTAACACTGCCATCCAAATAATCCCTTA<br>551 AATTGTCCAGGTTATTAATTCCCTGACCTGAAGGCAAATCTCTGGACTCC<br>601 CCTCCAGTGCCCTTTACATCCTCAAAAACTACTAAAAACTGGTCAATAGC<br>651 TACTCCTAGCTCAAAGTTCAGCCTGTCCAAGGGCAAATTAACATTTAAAG<br>701 CTTTCCCCCCACATAATTCAAGCAAAGCAGCTGCTAATGTAGTTTTACCA<br>751 CTATCAATTGGTCCTTTAAACAGCCAGTAGGAATGTTGTACACCATGCAT<br>801 TTTAAAAAGTCATACACCACTGAATCCATTTTGGGCAACAAACAGTGTAG<br>851 CCAAGCAACTCCAGCCATCCATTCTTCTATGTCAGCAGAGCCTGTAGAAC<br>901 CAAACATTATATCCATCCTATCCAAAAGATCATTAAATCTGTTTGTTAAC<br>951 ATTTGTTCTCTAGTTAATTGTAGGCTATCAACCCGTAAAACAGTATCAAC<br>1001 AGCCTGTTGGCATATGCTGTCAGCAAATATAGCAGCATTTGCATAATGCT<br>1051 TTTCATGGTACTTATAGTGGCTGGGCTGTATACATTTTAAACACATTTCA<br>1101 AAACTGTACTGAAATTCCAAGTACATCCCAAGCAATAACAACACATCATC<br>1151 ACATTGCATACTCTGTTACAAGCTTCCAGGACACTTGTTTAGTTTCCTCT<br>1201 GCTTCTTCTGGATTAAAATCATGCTCCTTTAACCCACCTGGCAAACTTTC<br>1251 CTCAATAACAGAAAATGGATCTCTAGTCAAGGCACTATACATCAAATATT<br>1301 CCTTATTAACCCCTTTACAAATTAAAAAGCTAAAGGTACACAATTTTTGA<br>1351 GCATAGTTATTAATAGCAGACACTCTATGCCTGTGTGGAGTAAAGTATGT<br>1401 TATGATTATAACTGTTATGCCTACTTATAAAGGTTACAGAATATTTTTCC<br>1451 ATAATTTTCTTGTATAGCAGTGCAGCTTTTTCCTTTGTGGTGTAAATAGC<br>1501 AAAGCAAGCAAGAGTTCTATTACTAAACACAGCATGACTCAAAAAACTTA<br>1551 GCAATTCTGAAGGAAAGTCCTTGGGGTCTTCTACGAGGAGTAGAATGTTG<br>1601 AGAGTCAGCAGTAGCCTCATCATCACTAGATGGCATTTCTTCTGAGCAAA<br>1651 ACAGGTTTTCCTCATTAAAGGCATTCCACCACTGCTCCCATTCATCAGTT<br>1701 CCATAGGTTGGAATCTAAAATACACAAACAATTAGAATCAGTAGTTTAAC<br>1751 ACATTATACACTTAAAAATTTTATATTTACCTTAGAGCTTTAAATCTCTG<br>1801 TAGGTAGTTTG**TCCAATTATGTCACACCACAGAAGTAAGGTTCCTTCACA**<br>1851 **AAGATCAAGTCCAAACCATCATTCTAAAGCAATCGAAGCAGTAGC**AATCA<br>1901 ACCCACACAAGTGGATCTTTCCTGTATAATTTTCTATTTTCATGCTTCAT<br>1951 CCTCAGTAAGCACAGCAAGCATATGCAGTTAGCAGACATTTTCTTTGCAC<br>2001 ACTCAGGCCATTGTTTGCAGTACATTGCATCAACACCAGGATTTAAGGAA<br>2051 GAAGCAAATACCTCAGTTGCATCCCAGAAGCCTCCAAAGTCAGGTTGATG<br>2101 AGCATATTTTACTCCATCTTCCATTTTCTTGTACAGAGTATTCATCTTCA<br>2151 TCTCCTCCTTTATCAGGATGAAACTCCTTGCATTTTTTT**AAATATGCCTT**<br>2201 **TCTCATCAGAGGAATATTCCCCCAGGCACTCCTTTCAAGACCTAGAAGGT**<br>2251 **CCATTAGCTGCAAAGATTCCTCTC**TGTTTAAAACTTTATCCAT
**E1A**	Adenoviral early region 1A gene	986	1<br>2	**45–113**<br>**874–940**	1 ATGAGACATATTATCTGCCACGGAGGTGTTATTACCGAAGAAAT**GGCCGC**<br>51 **CAGTCTTTTGGACCAGCTGATCGAAGAGGTACTGGCTGATAATCTTCCAC**<br>101 **CTCCTAGCCATTTT**TGAACCACCTACCCTTCACGAACTGTATGATTTAGAC<br>151 GTGACGGCCCCCGAAGATCCCAACGAGGAGGCGGTTTCGCAGATTTTTCC<br>201 CGACTCTGTAATGTTGGCGGTGCAGGAAGGGATTGACTTACTCACTTTTC<br>251 CGCCGGCGCCCGGTTCTCCGGAGCCGCCTCACCTTTCCCGGCAGCCCGAG<br>301 CAGCCGGAGCAGAGAGCCTTGGGTCCGGTTTCTATGCCAAACCTTGTACC<br>351 GGAGGTGATCGATCTTACCTGCCACGAGGCTGGCTTTCCACCCAGTGACG<br>401 ACGAGGATGAAGAGGGTGAGGAGTTTGTGTTAGATTATGTGGAGCACCCC<br>451 GGGCACGGTTGCAGGTCTTGTCATTATCACCGGAGGAATACGGGGGACCC<br>501 AGATATTATGTGTTCGCTTTGCTATATGAGGACCTGTGGCATGTTTGTCT<br>551 ACAGTAAGTGAAAATTATGGGCAGTGGGTGATAGAGTGGTGGGTTTGGTG<br>601 TGGTAATTTTTTTTTTAATTTTTACAGTTTTGTGGTTTAAAGAATTTTGT<br>651 ATTGTGATTTTTTTAAAAGGTCCTGTGTCTGAACCTGAGCCTGAGCCCGA<br>701 GCCAGAACCGGAGCCTGCAAGACCTACCCGCCGTCCTAAAATGGCGCCTG<br>751 CTATCCTGAGACGCCCGACATCACCTGTGTCTAGAGAATGCAATAGTAGT<br>801 ACGGATAGCTGTGACTCCGGTCCTTCTAACACACCTCCTGAGATACACCC<br>851 GGTGGTCCCGCTGTGCCCCATTA**AACCAGTTGCCGTGAGAGTTGGTGGGC**<br>901 **GTCGCCAGGCTGTGGAATGTATCGAGGACTTGCTTAACGA**GCCTGGGCAA<br>951 CCTTTGGACTTGAGCTGTAAACGCCCCAGGCCATAA

Table S4: Platform-specific definitions of LOB, LOD and LOQ in digital PCR and countable PCR.

Assay Performance characteristic		Definition	Digital PCR	Countable PCR
**Limit of blank**	LOB	Highest expected signal from blank samples	NA	LOB = mean of blank reactions + 1.645 × SD of blank reactions
**Limit of detection**	LOD	Lowest signal level that can be distinguished from background	Minimum copy number concentration reliably detected, defined as all technical replicates being positive (≥3 positive droplets)	LOD = LOB + 1.645 × SD of reactions targeting 10 counts per reactions
**Limit of quantification**	LOQ	Lowest target level that can be quantified with predefined precision criteria	Mean copy number per reaction at the lowest target level where %CV < 20% across technical replicates	LOQ = Mean counts per reaction at the lowest target level where %CV ≤ 20% across technical replicates

Table S5: Quantitative bias between expected and measured copy numbers across the dilution series for each dPCR assay in different duplex combinations.

Target	dPCR assay combination	Expected copies/ rnx	Mean measured copies / rnx	%bias	Absolute % bias	Mean measured copies / rnx	%bias	Absolute % bias
			Assay 1 (or 2)			Assay 3		
Amp	1+3	80000	64882.0	-18.9	18.9	62358.7	-22.1	22.1
		10000	8972.0	-10.3	10.3	8108.5	-18.9	18.9
		2000	1789.4	-10.5	10.5	1606.7	-19.7	19.7
		1000	909.0	-9.1	9.1	815.7	-18.4	18.4
		100	89.6	-10.4	10.4	82.0	-18.0	18.0
		50	45.5	-9.0	9.0	40.1	-19.7	19.7
		20	20.1	0.7	0.7	18.4	-7.9	7.9
		10	9.5	-5.3	5.3	9.3	-7.2	7.2
	2+3	80000	66422.7	-17.0	17.0	62310.7	-22.1	22.1
		10000	8593.6	-14.1	14.1	8072.7	-19.3	19.3
		2000	1647.1	-17.6	17.6	1634.8	-18.3	18.3
		1000	881.5	-11.9	11.9	831.7	-16.8	16.8
		100	87.3	-12.7	12.7	83.1	-16.9	16.9
		50	44.5	-11.0	11.0	41.8	-16.4	16.4
		20	18.6	-7.1	7.1	17.2	-14.2	14.2
		10	11.1	10.9	10.9	10.5	4.7	4.7
Kan	1+3	80000	77253.3	-3.4	3.4	77309.4	-3.4	3.4
		10000	10190.6	1.9	1.9	10165.4	1.7	1.7
		2000	2104.7	5.2	5.2	2093.5	4.7	4.7
		1000	1017.8	1.8	1.8	1001.9	0.2	0.2
		100	110.0	10.0	10.0	106.2	6.2	6.2
		50	53.2	6.5	6.5	52.9	5.8	5.8
		20	22.4	11.9	11.9	21.5	7.7	7.7
		10	11.0	9.7	9.7	9.6	-3.6	3.6
	2+3	80000	73875.4	-7.7	7.7	77807.6	-2.7	2.7
		10000	9446.3	-5.5	5.5	10047.7	0.5	0.5
		2000	1906.5	-4.7	4.7	2027.5	1.4	1.4
		1000	932.2	-6.8	6.8	985.0	-1.5	1.5
		100	93.3	-6.7	6.7	95.3	-4.7	4.7
		50	49.6	-0.9	0.9	54.1	8.2	8.2
		20	21.7	8.4	8.4	22.2	10.9	10.9
		10	10.4	3.6	3.6	10.3	3.4	3.4
SV40T	1+3	80000	67672.4	-15.4	15.4	75993.5	-5.0	5.0
		10000	8503.9	-15.0	15.0	9532.9	-4.7	4.7
		2000	1747.7	-12.6	12.6	1963.4	-1.8	1.8
		1000	871.6	-12.8	12.8	980.1	-2.0	2.0
		100	96.8	-3.2	3.2	109.6	9.6	9.6
		50	44.3	-11.5	11.5	51.3	2.7	2.7
		20	20.0	0.0	0.0	21.9	9.5	9.5
		10	10.0	-0.4	0.4	12.3	23.2	23.2
	2+3	80000	70729.3	-11.6	11.6	78282.1	-2.1	2.1
		10000	9189.7	-8.1	8.1	9896.1	-1.0	1.0
		2000	1875.2	-6.2	6.2	2041.0	2.0	2.0
		1000	901.5	-9.9	9.9	973.6	-2.6	2.6
		100	101.9	1.9	1.9	111.0	11.0	11.0
		50	53.3	6.5	6.5	56.8	13.7	13.7
		20	19.0	-5.0	5.0	21.0	5.1	5.1
		10	11.6	16.2	16.2	11.8	17.7	17.7
E1A	1+2	80000	85173.5	6.5	6.5	89772.5	12.2	12.2
		10000	11091.2	10.9	10.9	11681.8	16.8	16.8
		2000	2251.2	12.6	12.6	2379.1	19.0	19.0
		1000	1097.3	9.7	9.7	1150.9	15.1	15.1
		100	121.4	21.4	21.4	127.2	27.2	27.2
		50	59.8	19.7	19.7	61.7	23.4	23.4
		20	23.6	18.0	18.0	19.4	-3.1	3.1
		10	14.3	43.4	43.4	14.7	46.8	46.8

Table S6: Restriction digest reactions details.

gBlock used	Reaction name	Restriction enzyme used	Units of the enzyme used in reaction	Buffer used	Incubation time (min) and temperature (°C)	Inactivation time and temperature (°C)	Expected number of fragments	Expected lengths of fragments (bp)
Amp	BaeGI	BaeGI	10	NEBuffer r3.1(New England Biolabs, NEB)	15<br>37	20<br>80	2	120<br>741
	AseI	AseI	10				2	248<br>613
	BaeGI+AseI	BaeGI<br>AseI	10<br>10				3	120<br>248<br>493
	H_2O	/	/				1	861
Kan	AseI	AseI	10				2	183<br>633
	Bpu10I	Bpu10I	5				2	364<br>452
	AseI+Bpu10I	AseI<br>Bpu10I	10<br>5				3	181<br>183<br>452
	H_2O	/	/				1	816
mSV40T	BaeGI	BaeGI	10				2	611<br>1682
	BstXI	BstXI	10				2	383<br>1910
	BaeGI+BstXI	BaeGI<br>BstXI	10<br>10				3	383<br>611<br>1299
	H_2O	/	/				1	2293
E1A	SmaI	SmaI	20	rCutSmart Buffer (New England Biolabs, NEB)	15<br>37	20<br>65	2	450<br>536
	H_2O	/	/				1	986

Table S7: Detailed capillary electrophoresis results showing the presence of unfragmented gBlocks or singly restricted gBlocks observed in the double-restriction reactions.

gBlock	Restriction enzyme used	Restriction location (A. between target 1 and 2, B. between targets 2 and 3).	% of gBlocks cleaved only at A	% of gBlocks cleaved only at B	% of unfragmented gBlocks
**Amp**	BaeGI+AseI	A&B	1.4	2.0	1.0
**Kan**	AseI+Bpu10I	A&B	17.6	0	2.1
**mSV40T**	BaeGI+BstXI	A&B	5.0	2.7	1.8

Table S8: Detailed performance characteristics of Countable PCR for ampicillin (Amp) and E1A assays' targets across a range of target counts per reaction.

	Amp						E1A			
	1.		2.		3.		1.		2.	
**Targeted counts per reaction**	Mean number of counts per reaction	%CV	Mean number of counts per reaction	%CV	Mean number of counts per reaction	%CV	Mean number of counts per reaction	%CV	Mean number of counts per reaction	%CV
0	2	80.8	2	127.3	4	50.8	2	94.4	3	78.5
10	11	35.5	8	33.7	10	37.6	12	42.9	12	38.5
20	23	27.9	19	27.2	18	38.0	21	43.1	24	33.6
50	52	20.0	42	20.6	43	21.2	51	22.8	55	17.1
60	60	21.3	50	25.2	48	26.8	57	18.4	56	22.6
75	78	18.6	67	18.5	71	46.9	65	17.4	63	29.1

Table S9: Summary of nanopore sequencing quality metrics.

Fraction	DNase pretreatment	Barcode	Number of reads	Mean read length (bp)	Median read length (bp)	Read length N50	Mean Q-score
**Heavy**	+	1	792495	2109.8	1290	4270	11.7
	-	2	1011054	1425.7	670	3557	11.9
**Full**	+	3	579458	2584.0	1888	4274	11.5
	-	4	842833	1917.0	1197.0	4251	11.6
**Intermediate**	+	5	648546	2571.1	1872.0	4268	11.6
	-	6	712012	2455.0	1841.0	4263	11.5
**Empty**	+	7	703046	2580.7	1876.0	4264	11.6
	-	8	675772	2537.9	1871.0	4264	11.5

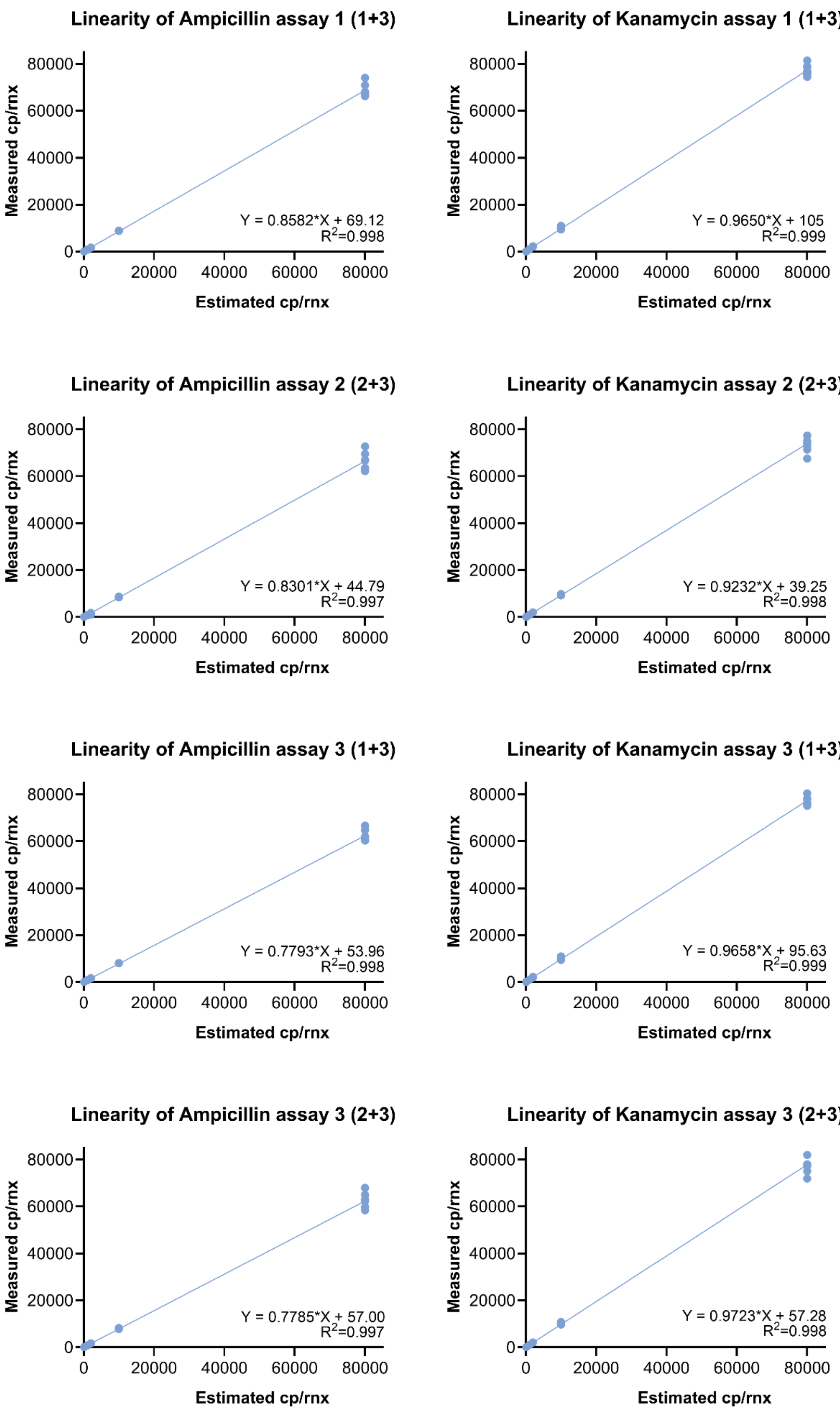

Figure S1: Determination of linearity for Ampicillin and Kanamycin digital PCR (dPCR) assays tested in double-duplex reaction setups. Assays 1 and 2 were used interchangeably in combination with assay 3. Linearity was determined for each assay in both duplex configurations.

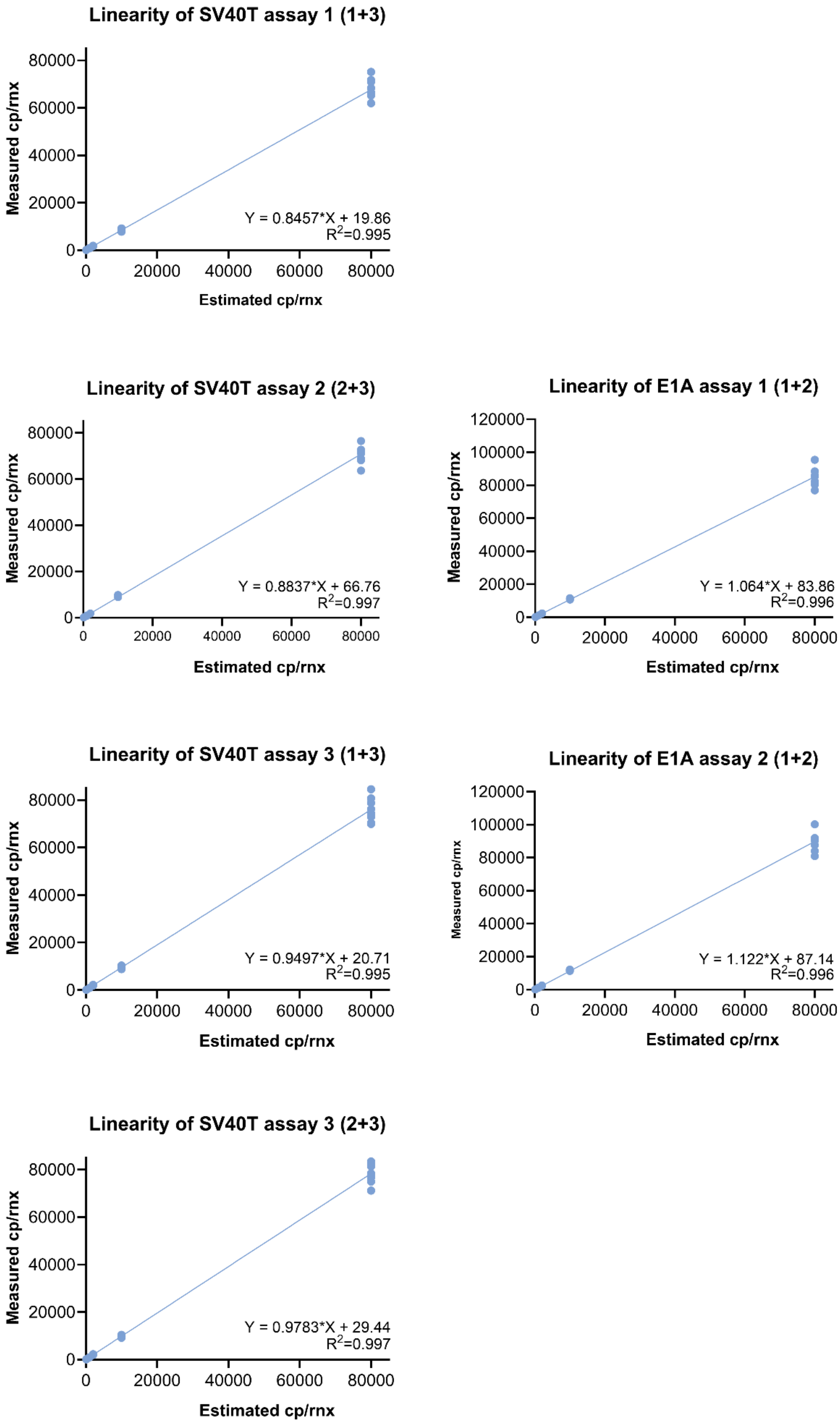

Figure S2: Determination of linearity for SV40T and E1A digital PCR (dPCR) assays tested in duplex reaction setups. Assays 1 and 2 were used interchangeably in combination with assay 3 in SV40T assay testing. Linearity was determined for each assay in both duplex configurations. E1A testing consisted of two targets only in one duplex setup.

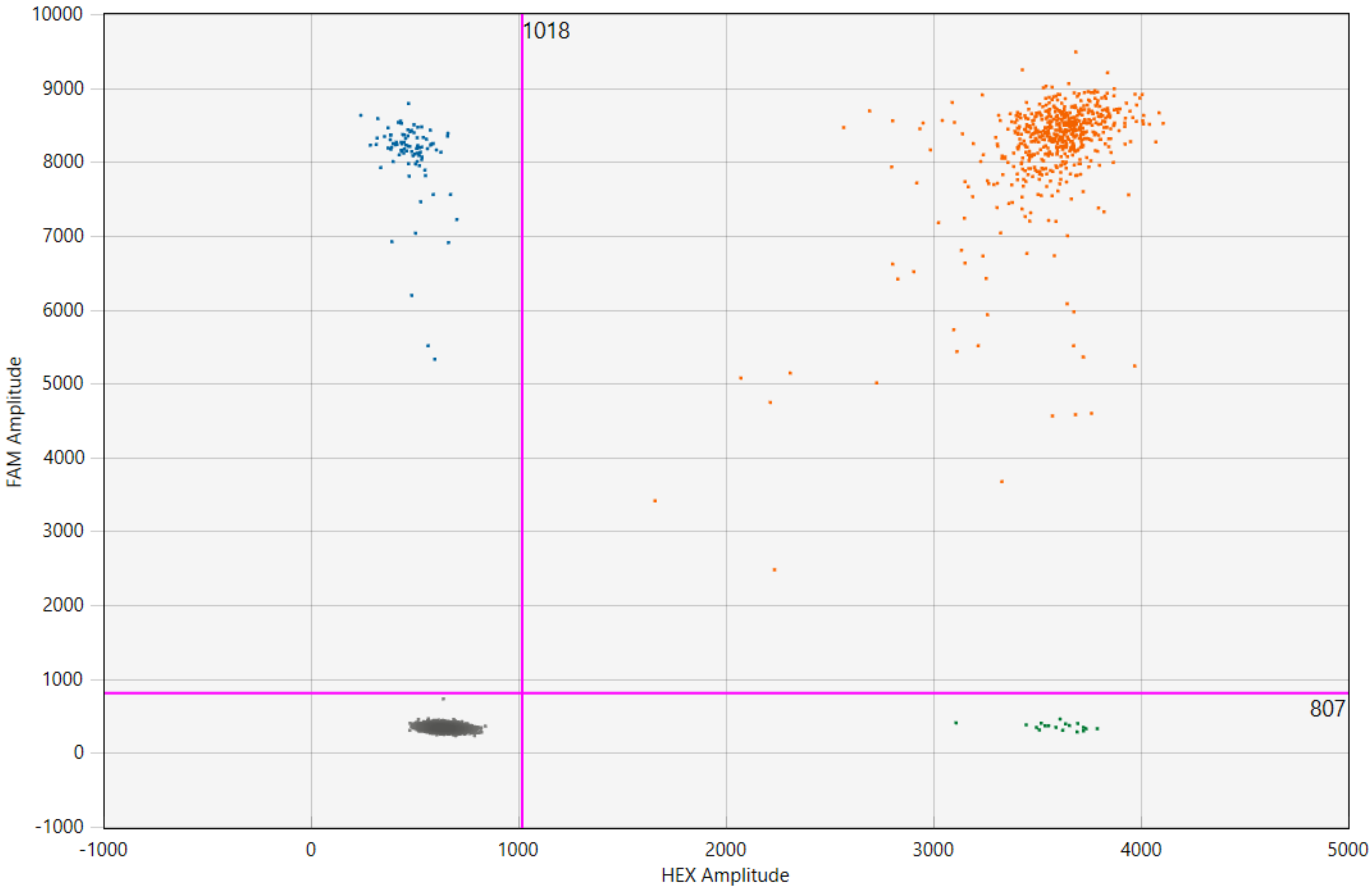

Figure S3: The 2D digital PCR plot of the unfragmented ampicillin gBlock using duplex assays. Representative two-dimensional (2D) digital PCR plot showing simultaneous detection in both fluorescence channels for the duplex assay. Assay 1 is labelled with FAM and assay 3 with HEX.

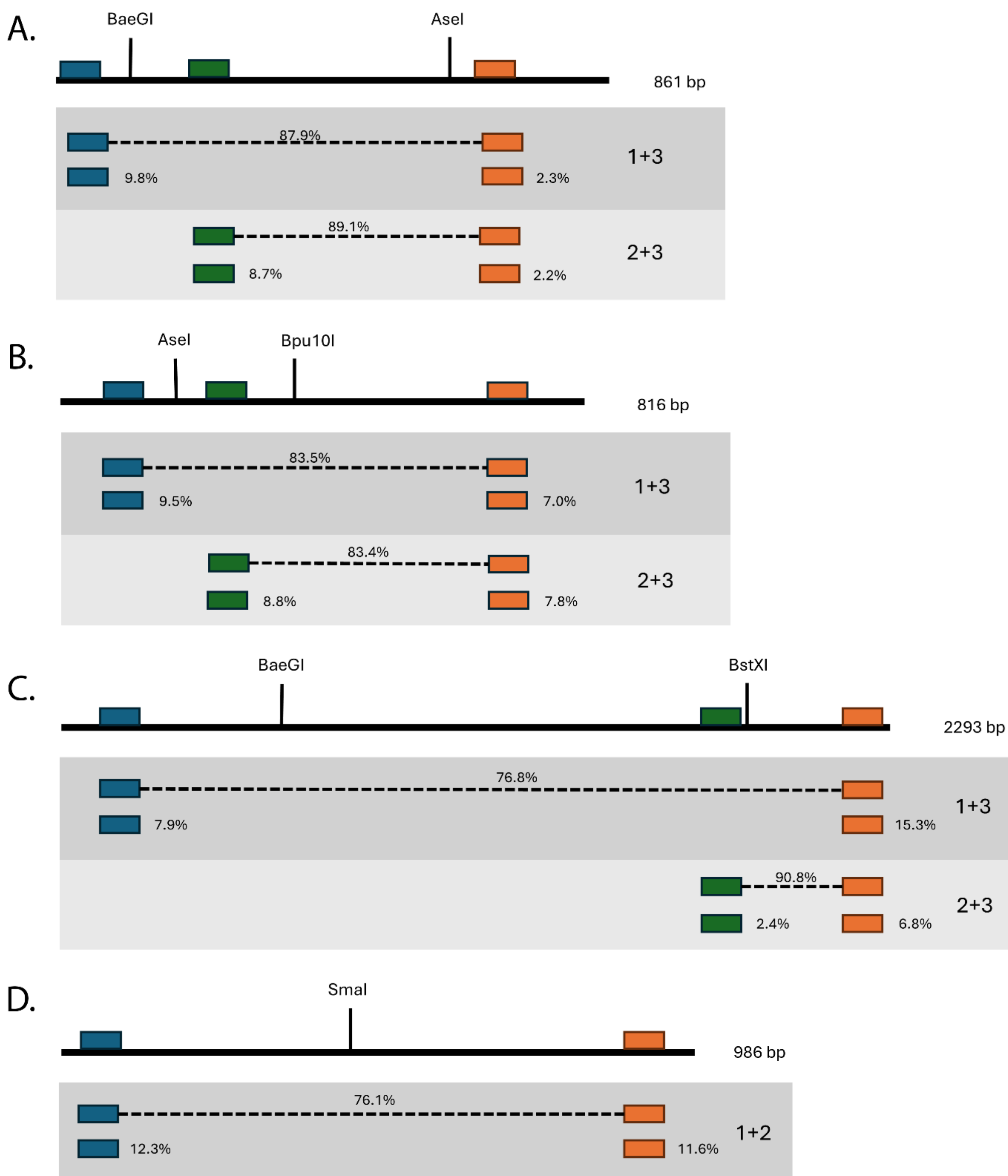

Figure S4: Detail dPCR results of unfragmented gBlocks testing. (A) Ampicillin gBlock. (B) Kanamycin gBlock. (C) mSV40T gBlock, (D) E1A gBlock. Minor deviations from 100% are attributable to rounding of individual values.

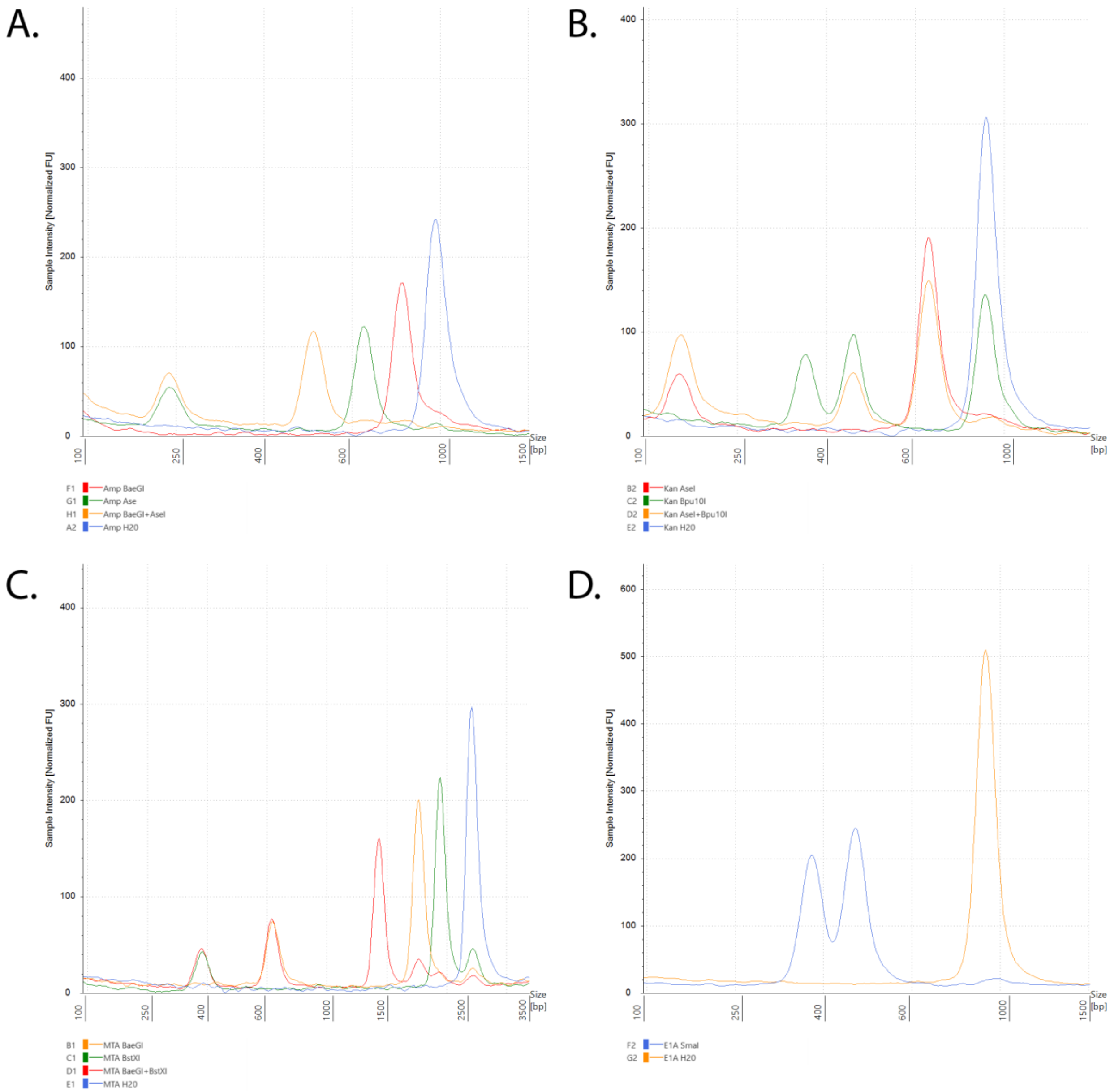

.

Figure S5: Restriction digest analysis of gBlocks by TapeStation. Electropherograms generated using TapeStation Analysis Software after restriction digestion of gBlocks with various enzymes (e.g., BaeGI, AseI, or a combination of BaeGI and AseI). Samples labeled "H_2O" represent undigested controls, where water was added instead of restriction enzyme. (A) Ampicillin gBlock, (B) Kanamycin gBlock, (C) mSV40T gBlock (named as MTA), and (D) E1A gBlock.

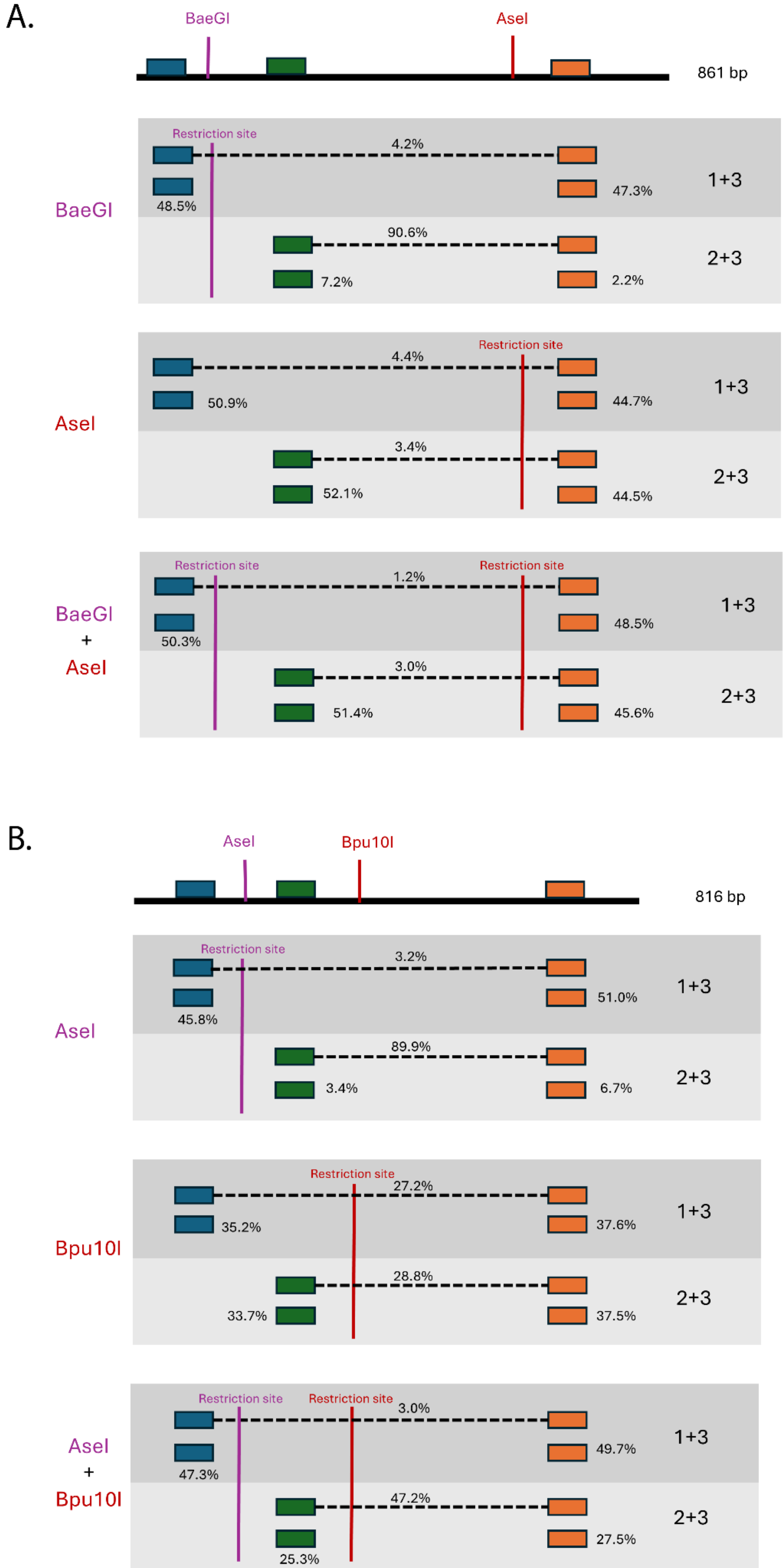

Figure S6: Detail dPCR results of restricted Ampicillin (A.) and Kanamycin (B) gBlocks testing. Ampicillin gBlock was digested with BaeGI or AseI, as well as with both enzymes simultaneously. Kanamycin gBlock was digested with AseI or Bpu10I, as well as with both enzymes. Minor deviations from 100% are attributable to rounding of individual values.

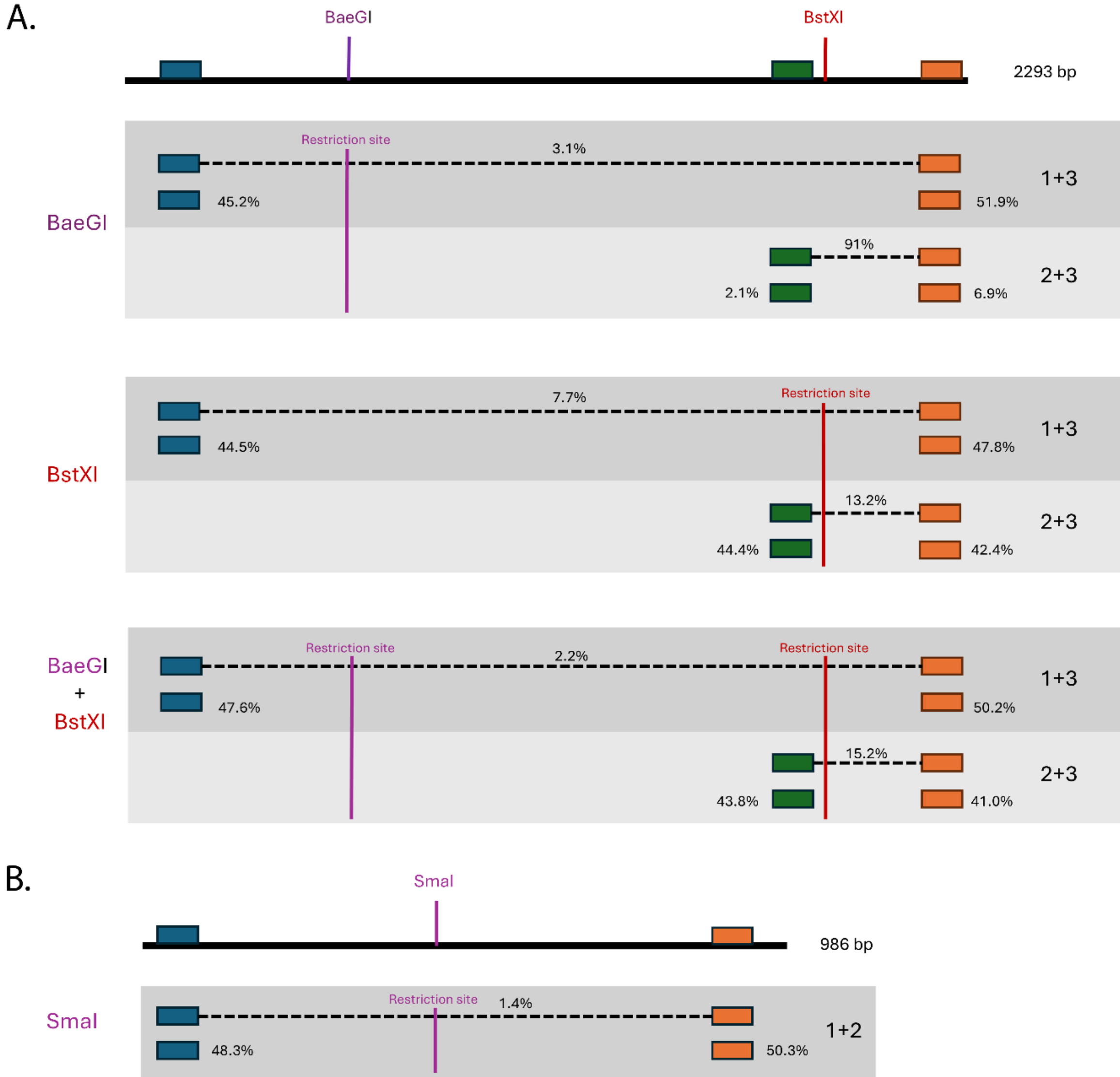

Figure S7: Detail dPCR results of restricted mSV40T (A) and E1A (B) gBlocks testing. mSV40T gBlock was digested with BaeGI or BstXI, as well as with both enzymes. E1A gBlock was digested with SmaI. Minor deviations from 100% are attributable to rounding of individual values.

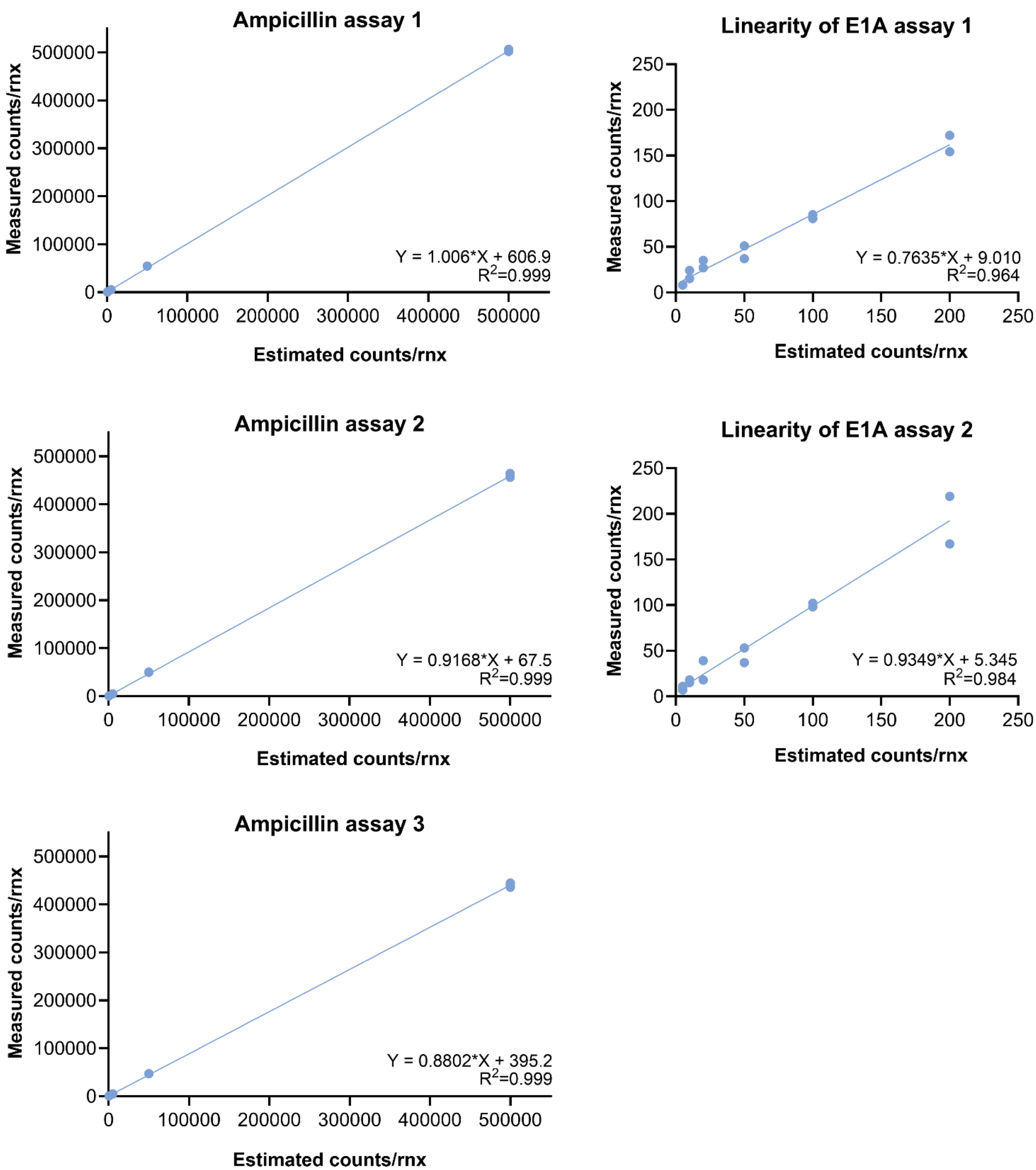

Figure S8: Linearity of countable PCR (cPCR) assays across dilution series, showing the relationship between expected and measured copies per reaction.

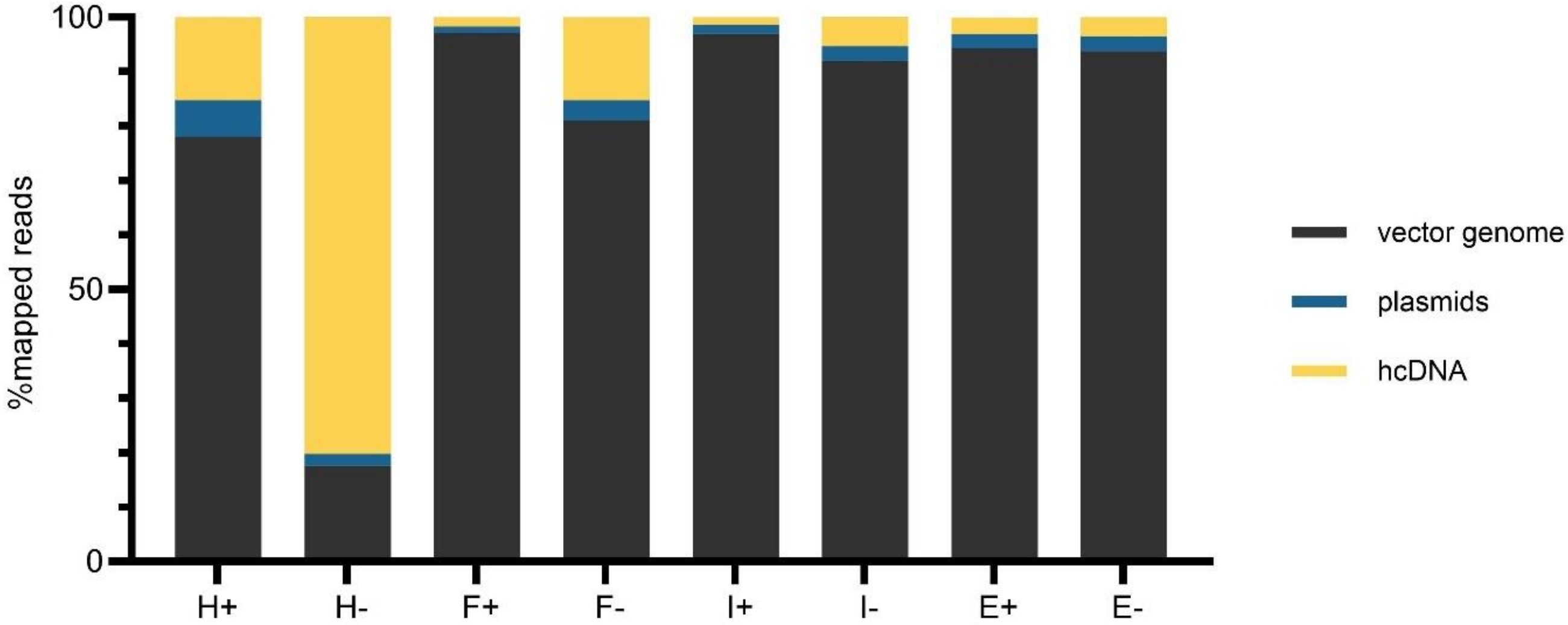

Figure S9: Nanopore read mapping results expressed as the % of total Nanopore sequencing reads mapping to either vector genome, plasmids or host cell DNA for each DNase I treated (+) and untreated sample (-). H = heavy, F = full, I = intermediate and E = empty fraction.

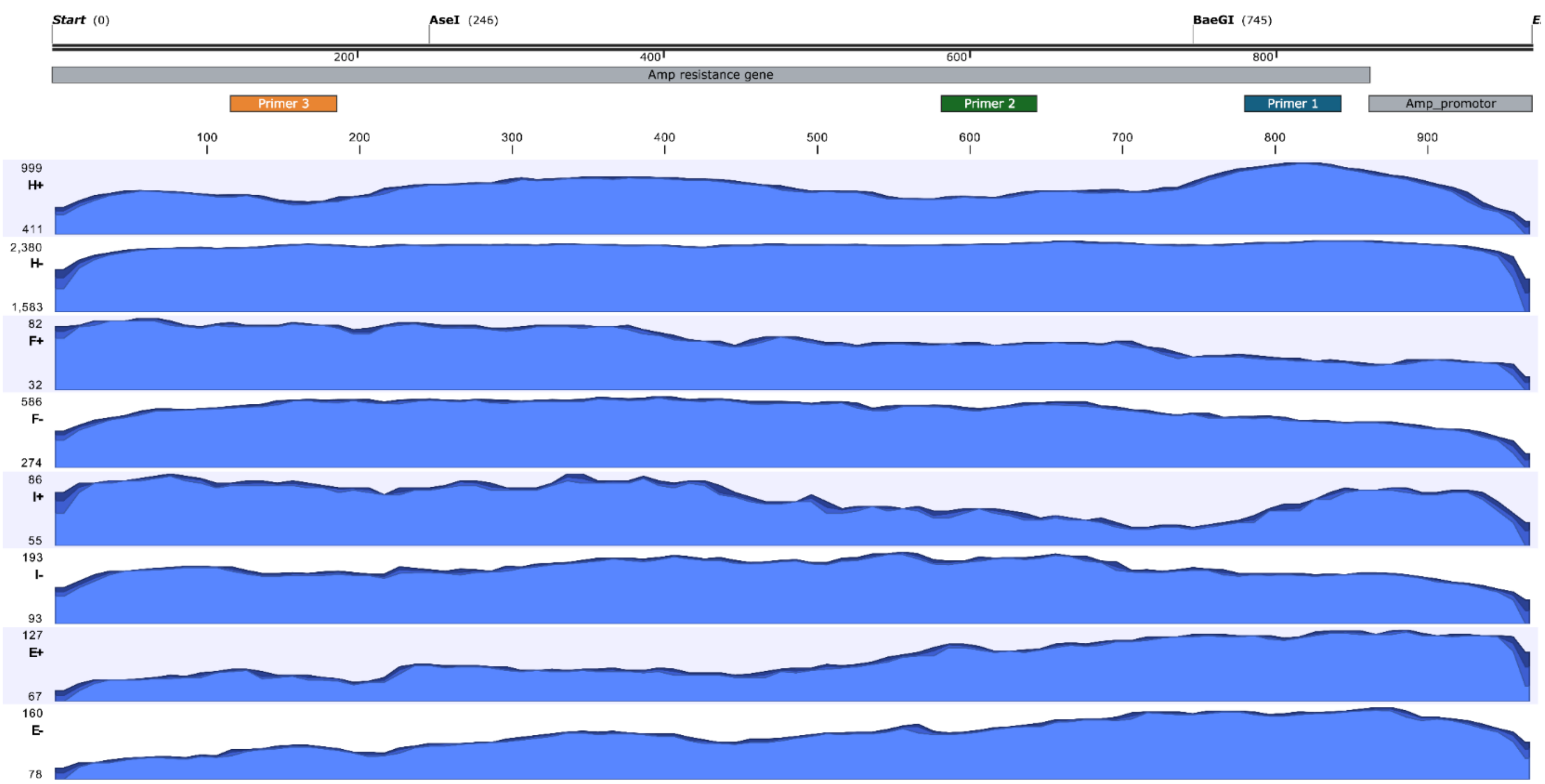

Figure S10: Coverage profiles of the ampicillin resistance gene in DNase I–treated (+) and untreated (–) fractions: heavy (H), full (F), intermediate (I), and empty (E). The top panel illustrates the reference genome containing the ampicillin resistance gene and its promoter, along with the genomic coordinates of the digital PCR assays developed in this study. Coverage tracks below represent read depth across the sequence. The x-axis indicates nucleotide position (bp), and the y-axis indicates read depth (number of reads mapped to each nucleotide position, linear scale). The values shown on the left of each track represent the minimum and maximum read depth observed within each sample.
