# Supplementary material for: Development and qualification of quantitative assays for detecting process-related nucleic acid impurities in recombinant AAV preparations
Source: Mol Ther Adv. 2026 Sep 30;34(4):201854. doi: 10.1016/j.omta.2026.201854 (PMC13720811; doi:10.1016/j.omta.2026.201854)
Supplement: Supplementary file 2 [file mmc2.pdf]

Molecular Therapy
Advances
Original Article

Development and qualification of quantitative assays for detecting process-related nucleic acid impurities in recombinant AAV preparations

Mojca Janc[1,2] and David Dobnik[1,3]

[1]National Institute of Biology, Večna Pot 121, 1000 Ljubljana, Slovenia; [2]Jožef Stefan International Postgraduate School, Jamova Cesta 39, 1000 Ljubljana, Slovenia; [3]NibaLabs D.o.o., Litostrojska 52, 1000 Ljubljana, Slovenia

Residual DNA impurities in recombinant adeno-associated virus (rAAV) vector preparations pose an important analytical and regulatory challenge, yet current methods provide limited information on their integrity. Here, we developed multiplex digital PCR (dPCR) workflows that quantitatively distinguish full-length from fragmented DNA impurities originating from plasmid backbones (ampicillin and kanamycin resistance genes) and host-cell DNA (adenoviral E1A and SV40 large T antigen). By strategically positioning terminal and internal assays across each target, the method enables integrity assessment beyond the capabilities of other methods. In our CsCl-purified scAAV9 preparations, plasmid-derived sequences were abundant and largely DNase I resistant, confirming their encapsidation, whereas host-cell-derived impurities were present at low levels and mostly removed by nuclease treatment. Obtained dPCR results were further confirmed using countable PCR, a recently introduced single-molecule quantification technology, and with nanopore sequencing. Moreover, countable PCR platform was shown to be less sensitive to the highly concentrated AAV background, when quantifying rare targets. Overall, our results demonstrate that multiplex dPCR is an informative tool for integrity profiling of impurities in rAAV manufacturing.

INTRODUCTION

Rapid developments in biomedicine and viral biology have established viral vectors for diverse applications from vaccines to gene and cell therapy. Adeno-associated viruses (AAVs) are particularly valued due to low immunogenicity, no pathogenicity, minimal integration, and broad mammalian tropism.[1,2]

Recombinant AAV (rAAV) vectors are generated by replacing the rep and cap genes from AAVs with promoter-driven gene of interest (GOI), while retaining only the viral inverted terminal repeats (ITRs), which are essential for replication and packaging.[3] Production of rAAV typically relies on transient transfection of HEK293-derived cells with separate plasmids carrying rep/cap functions and helper genes from the helper virus (e.g., adenoviral E1A, E2A, E4, and VA RNA) or use of stable cell lines (different production approaches are reviewed by others[4,5]). The helper functions of E1A and SV40 large T antigen (SV40T) on the production of rAAV are well understood.[6] Most importantly, E1A activates the AAV p5 and p19 promoters that control the expression of AAV Rep proteins, while SV40T further enhances helper function by stimulating Rep protein activity. In addition to their essential role in the production, these helper genes are also well characterized cell-transforming elements: E1A promotes uncontrolled cell cycle progression, while SV40T disrupts tumor suppressor pathways, including p53 and Rb, and are considered potential oncogenes.[7,8]

Although the main goal of the rAAV production is to produce viral particles exclusively packed with the complete expression cassette that is not always achieved.[9–12] In addition to truncated vector genomes, unintended encapsidation of residual DNA has been widely reported. Such contaminants include potential oncogenes (e.g., E1A and SV40T) and plasmid-derived antibiotic resistance genes, such as ampicillin and kanamycin, originating from plasmid backbones used during vector production.[9–20] Although antibiotic resistance genes lack mammalian promoters, those sequences can persist *in vivo* following AAV injection.[19–21] ITR regions originating from the plasmid backbone may trigger their packaging and transcription if a complete reading frame is present.[19–21] Essential monitoring of those DNA impurities not only addresses the safety considerations but also serves as an indicator of successful purification performance and DNase I treatment effectiveness.

Given the quality control need, the rAAV workflows rely on several analytical methods, with quantitative PCR (qPCR) and digital PCR (dPCR) being the most often used approaches due to their high sensitivity, specificity, and routine in-process monitoring.[14,16–19] While commercial kits (e.g., the resDNA SEQ qPCR and dPCR from Thermo Fisher Scientific) offer convenient solutions for

Received 3 March 2026; accepted 28 September 2026;
https://doi.org/10.1016/j.omta.2026.201854.

Correspondence: Mojca Janc, Department of Biotechnology and Systems Biology National Institute of Biology, Večna pot 121, Ljubljana 1000, Slovenia.
E-mail: mojca.janc@nib.si

© 2026 The Authors.
Published by Elsevier Inc. on behalf of The American Society of Gene and Cell Therapy.
This is an open access article under the CC BY license (http://creativecommons.org/licenses/by/4.0/).

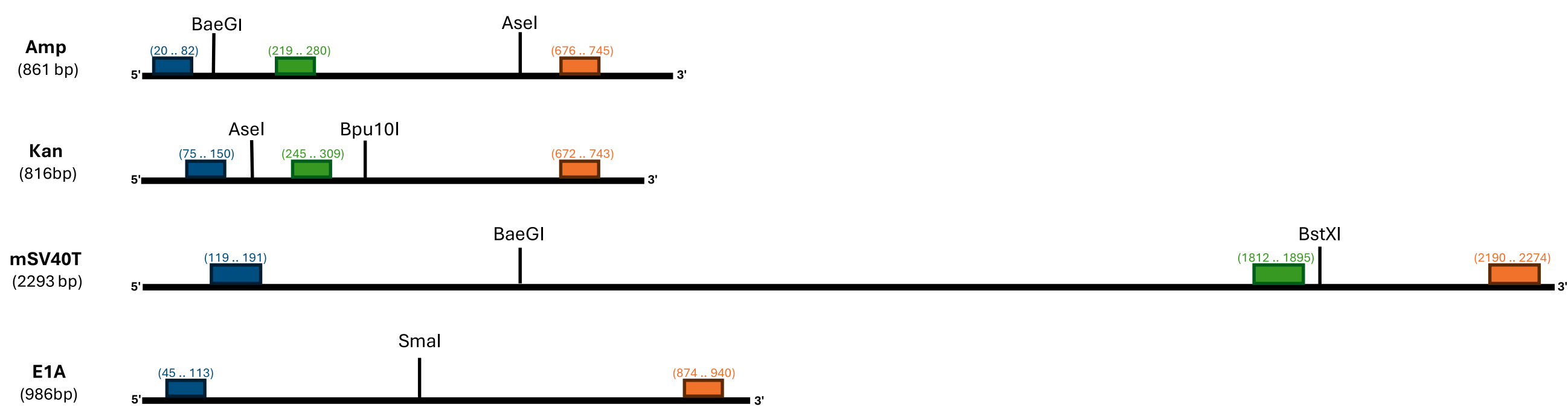

Figure 1. DNA impurity targets

Schematic representation showing the positions of newly developed dPCR assays (blue arrows indicate 1st FAM-labeled assays, green arrows indicate 2nd FAM-labeled assays and orange arrow indicates HEX-labeled assay) and the locations of restriction enzymes applied during assay development. Amp, ampicillin resistance gene; Kan, kanamycin resistance gene; SV40T, SV40 large T antigen; E1A, adenoviral E1A gene.

host-cell or plasmid DNA quantification, developing in-house assays, as in this study, provides greater flexibility, transparency, and cost-efficiency, which is particularly advantageous for research laboratories. High-throughput sequencing platforms, including short- and long-read sequencing, provide comprehensive profiling of residual DNA, enabling detection of both expected and unexpected contaminants and assessment of fragment size distribution.[10–14,20]

In this study, we developed and qualified sensitive duplex dPCR assays for the detection and quantification of full-length DNA impurities originating from the rAAV production process. The assays were designed to target complete antibiotic resistance genes (ampicillin and kanamycin) derived from the production plasmids, as well as HEK293T cell-associated host-cell contaminations, complete SV40T, and adenoviral E1A gene. The performance of these assays was assessed using synthetic DNA and extracted genomic DNA and subsequently applied to representative rAAV preparations. In addition, the hydrolysis-probe assays were adapted to the universal multiplexing (UM) chemistry and evaluated on the countable PCR platform (Countable Labs) as an orthogonal PCR approach.[22,23] This platform was included to address analytical limitations commonly seen in droplet-based multiplex dPCR, particularly when quantifying low-abundance targets. This work aims to provide comprehensive solutions for characterization of residual DNA in rAAV preparations, by different assays targeting complete genes, and aims to support improved analytical control of vector purity during process development and quality assessment.

RESULTS

Development and qualification of multiplex dPCR assays

We designed distinct dPCR primer and probe combinations (also referred to as dPCR assays) for each DNA impurity to assess their full-length presence (Figure 1). We targeted antibiotic resistance genes (ampicillin and kanamycin) originating from plasmid DNA, as well as potential oncogenes (adenoviral E1A gene and SV40T) derived from HEK293 host cell DNA. Details of the assay designs and dPCR workflow are provided in the methods, and in Tables S1−S3. By positioning targets at both ends and one within the internal region of each target gene, we were able to determine their full-length and shorter fragment concentration.

We first determined the linearity, limit of detection (LOD), and limit of quantification (LOQ) for each of the developed assays using four different gBlocks, one for each DNA impurity (Tables 1 and S4). LOD, LOQ, and linearity were determined from four independent dilution series of each gBlock, each comprising eight dilution points ranging from 10 to 8E+04 copies per reaction. In two separate duplex setups, assays 1 and 2, both labeled with FAM dye, were used interchangeably and paired with assay 3, labeled with HEX dye. The relation between expected dPCR copy number and the measured dPCR copy number concentration was linear ($R^2 > 0.99$) for all tested assays (Table 1; Figures S1 and S2). The LODs for all developed assays ranged from 9.3 to 21.9 copies per reaction, whereas the LOQs ranged from 17.2 to 59.8 copies per reaction. Agreement between expected and measured values was further evaluated by calculating % bias and absolute % bias across the dilution series (Table S5). Most assay-target combinations showed moderate deviations, typically within ±20% bias, although larger deviations were observed in specific configurations, particularly at lower copy numbers.

Interestingly, we observed single-channel-positive droplets on 2D readouts in all our duplex dPCR reactions while using gBlock to determine assays characteristics (Figures S3 and S4). This suggested that the original gBlocks were already fragmented upon receipt. Based on assays 1 and 3, we determined that the longest mSV40T gBlock had 76.8% full-length sequences, and 90.8% (shorter fragment) when targeted with assays 2 and 3. The percentage of full-length E1A gBlock was around 76.1%, while the ampicillin and kanamycin gBlocks were about 87.9% and 83.5%, respectively, when targeted with assays 1 and 3, but slightly less than 90% full-length (shorter fragment) when targeted with duplex assays 2 and 3. TapeStation analysis revealed a distinct peak in original gBlock sample (also referred as a H_2O sample in following restriction digest reaction) corresponding to the expected full-length gBlock, indicating

Table 1. Determined LOQ, LOD, LOB, and R^2 values for developed assays and corresponding targets on digital and countable PCR platforms

	Amp				E1A		Kan				SV40T			
dPCR assay (duplex combination)	1. (1 + 3)	2. (2 + 3)	3. (1 + 3)	3. (2 + 3)	1. (1 + 2)	2. (1 + 2)	1. (1 + 3)	2. (2 + 3)	3. (1 + 3)	3. (2 + 3)	1. (1 + 3)	2. (2 + 3)	3. (1 + 3)	3. (2 + 3)
LOQ mean cp/rnx	45.5	18.6	40.1	17.2	59.8	25.7	22.4	21.5	21.7	22.2	44.3	51.3	53.3	56.8
LOD mean cp/rnx	9.5	11.1	9.3	10.5	14.3	14.7	11.0	9.6	10.4	10.3	10.0	21.9	19.0	11.8
R^2	0.998	0.997	0.998	0.997	0.996	0.996	0.999	0.998	0.999	0.998	0.995	0.997	0.995	0.997
Countable PCR assay (multiplex combination)	1. (1 + 2+3)	2. (1 + 2+3)	3. (1 + 2+3)		1. (1 + 2)	2. (1 + 2)								
LOQ mean count/ rnx	52	67	>71		57	55	[a]							
LOD mean count/ rnx	10	11	14		14	15								
LOB mean count/rnx	4	7	8		6	8								
R^2	0.999	0.999	0.999		0.964	0.984								

Amp, ampicillin resistance gene; E1A, adenoviral E1A gene; Kan, kanamycin resistance gene, SV40T, SV40 large T antigen.
[a]Analysis not performed because the respective assay could not be implemented on the countable PCR platform, without further extensive optimization or re-design.

successful synthesis; however, the presence of a non-linear baseline and additional smaller peaks suggests that shorter DNA fragments may also be present in the samples (Table 2; Figure S5).

Next, gBlocks were digested using various restriction enzymes to either prepare individual fragments or to separate targets 1 and 2, while keeping targets 2 and 3 together (Table S6). TapeStation analysis indicated that the enzymatic digestion of gBlocks may not have been fully efficient, as fragments of the original size were still detectable alongside those of the predicted lengths (Table 2; Figure S5). Additionally, in the restriction digest reaction using two enzymes, we detected fragments of the original gBlocks that were cleaved by only one of the restriction enzymes rather than both (Table S7). Newly developed dPCR assays confirmed the presence of different fragments in these inefficient restriction digest reactions, demonstrating high sensitivity in detecting distinct fragment distributions (Table 2; Figures S6 and S7). These experiments showed that multiplex dPCR can detect full-length and also fragmented molecules that might be below the sensitivity limit of capillary electrophoresis, illustrating its sensitivity to mixed fragment populations.

Following initial testing of newly developed dPCR assays targeting HEK host cell-derived DNA impurities on various gBlocks, we also evaluated their performance on genomic DNA extracted from HEK293T cells (Table 3). The copy number per mL of the original sample was 3.8E+07 for the E1A gene, which was present in almost 90% full-length fragments. In contrast, the SV40T was detected at lower concentrations (ranging from 5.8E+06 to 8.6E+06) and only about 60% of the fragments were full-length.

Adaptation of dPCR assays into a real triplex using countable PCR platform

To further explore whether increased multiplexing could enhance full-length detection efficiency, hydrolysis-probe assays were adapted to UM chemistry, enabling true triplex reactions, and their performance was evaluated on the countable PCR platform. Initial runs following direct transfer from dPCR to the countable PCR platform, using the same gBlocks, extracted host cell DNA and AAV samples, were successful for the ampicillin and E1A targets. However, the kanamycin and SV40 T antigen assays showed suboptimal performance, which would require substantial further optimization, and were thus excluded from this study.

The LOD and LOQs for countable PCR were determined based on platform-specific criteria, as described in methods (Table 1; Table S4). For the ampicillin assays in triplex reactions for assays 1, 2, and 3, the LODs were 10, 11, and 14 counts per reaction (start, middle, and end target), respectively, while the LOQ were 52, 67, and above 71 counts per reaction, respectively. The LODs for E1A assays in duplex reactions were 14 and 15 counts per reaction, while LOQs were 57 and 55 counts per reaction. All ampicillin PCR assays demonstrated excellent linearity across the evaluated range, while E1A had slightly lower linearity values (Table 1; Figure S8; Table S8). Ampicillin assays showed broad dynamic range extending from 0 up to 500,000

Table 2. The proportion of unfragmented gBlocks following different restriction enzyme digestions assessed using capillary electrophoresis, digital PCR, and countable PCR platform

gBlock	Restriction enzyme used	Capillary electrophoresis % of Unfragmented gBlocks	Digital PCR (dPCR) % Unfragmented determined by duplex 1 + 3 dPCR	% Unfragmented determined by duplex 2 + 3 dPCR	Countable PCR % Linked molecules determined by multiplex
Amp	BaeGI	3.5	4.2	90.6	NA
	AseI	4.6	4.4	3.4	NA
	BaeGI+AseI	1.0	1.2	3.0	NA
	H_2O	100	87.9	89.1	65.9[c]
Kan	AseI	2.2	3.2	89.9	[d]
	Bpu10I	25.5	27.2	28.8	
	AseI+Bpu10I	2.1	3.0	47.2	
	H_2O	100	83.5	83.4	
mSV40T	BaeGI	3.8	3.1	90.9	[d]
	BstXI	8.0	7.7	13.1	
	BaeGI+BstXI	1.8	2.2	15.2	
	H_2O	100	76.8	90.8	
E1A	SmaI	1.6	1.4[a]		NA
	H_2O	100	76.1[a]		71.0[b]

NA, not assessed in this study.
[a]Values determined in duplex dPCR, where assays 1 and 2 were used.
[b]Values determined in duplex countable PCR, where assays 1 and 2 were used.
[c]Values determined in triplex countable PCR, where assays 1, 2, and 3 were used.
[d]Analysis not performed because the respective assay could not be implemented on the countable PCR platform, without further extensive optimization or re-design.

counts per reaction, reflecting the higher partition capacity of the countable PCR system. In contrast, the dynamic range for the E1A assay was evaluated only up to approximately 200 counts per reaction, as this target is not expected in AAV samples at high concentrations and therefore not relevant for method application.

Linkage analysis on this platform revealed a lower proportion of full-length gBlock and hcDNA fragments compared to dPCR workflow, with differences ranging from approximately 5%–23% (Tables 2 and 3). Using the UM-adapted assays, the proportion of full-length DNA impurities was estimated at 66% for the ampicillin gBlock, and 71% for the E1A gBlock, while extracted host cell DNA showed 80% full-length fragments.

Assessment of DNA impurities in scAAV9 samples

Newly developed dPCR assays were also tested on scAAV9, which was produced in adherent HEK293T cells and purified through consecutive CsCl ultracentrifugation runs. Heavy, full, intermediate, and empty fractions were previously described and characterized by our group.[24] This material does not represent a final pre-clinical or clinical-grade AAV product but was intentionally selected for method development and analytical assessment of residual DNA integrity, especially due to its impurity content.

Multiplex dPCR successfully detected the ampicillin resistance gene even in DNase I-treated rAAV samples (Table 4). Given previously determined viral genome titers in those fractions,[24] the ampicillin gene represented up to 3% of encapsidated genomes in DNase I-treated samples. The greatest reduction in ampicillin titer following DNase I pretreatment was observed in the heavy fraction (93%), whereas other fractions showed smaller reductions (up to 30% in the full fraction and up to 10% in the intermediate and empty fractions), suggesting that most ampicillin targets were encapsidated. Additionally, the highest percentage (58.9%) of full-length ampicillin resistance gene (determined in assay 1 and assay 3 multiplex reactions) was observed in DNase I untreated heavy fraction and the lowest (16.9%) in the treated empty fraction. Countable PCR performed on the untreated intermediate fraction revealed comparable titers across individual assays and linked molecules titer, supporting its use as an alternative method (Table 4).

In contrast to plasmid-derived impurities, the SV40T and E1A oncogenes were detected at substantially lower levels using dPCR workflow following DNase I treatment (data not shown). The nuclease treatment reduced their signal below the LOD. Without nuclease treatment of the samples, we were able to detect HEK host-cell-derived oncogenes using dPCR; however, in several cases they could not be reliably quantified, as the measured values fell below the LOQ (Table 5). We were able to quantify SV40T in the heavy fraction using assays 1 and 3, but not with the combination of assays 2 and 3. The number of positive droplets per reaction was close to the determined LOQ, which is higher for assay 2 than for assay 1. We determined that 7.2% of both signals correspond to full-length oncogenes. In the full fraction, the SV40T titer was determined to be 1.3E+05

Table 3. Evaluation of SV40 large T antigen and E1A in extracted HEK293T genomic DNA using digital PCR and countable PCR platform

	SV40 T antigen						E1A		
	Titer (*N* of target/mL)						Titer (*N* of target/mL)		
	Assay 1. (1 + 3)	Assay 2. (2 + 3)	Assay 3 (1 + 3)	Assay 3 (2 + 3)	% Linked molecules in 1 + 3 dPCR multiplex reaction	% Linked molecules in 2 + 3 dPCR multiplex reaction	Assay 1. (1 + 2)	Assay 2. (1 + 2)	% Linked molecules
Digital PCR	8.6E+06	6.0E+06	5.8E+06	6.0E+06	60.2	91.0	3.8E+07	3.8E+07	89.7
Countable PCR	[a]						1.8E+07	1.8E+07	80.4

Titers were determined separately for each developed assay, and the proportion of unfragmented SV40 large T antigen and E1A was calculated using only dilutions above the LOQ.
[a]Analysis not performed because the respective assay could not be implemented on the countable PCR platform, without further extensive optimization or re-design.

copies/mL; however, titers could not be determined with the other assays, indicating that SV40T sequence is most likely present in fragments rather than in full length. For assay 1, SV40T signals in the intermediate fraction were between the LOQ and LOD, whereas signals for all other assays in the intermediate and empty fractions were below the LOD, suggesting that SV40T is present at low levels.

Using the duplex dPCR assay (combination of 1 and 2) targeting the E1A gene, we detected E1A in all four fractions; however, we were unable to accurately determine the titer in the intermediate and empty fractions with assay 1 (Table 5). The titers of individual targets were approximately 2 times lower in full fraction than in heavy; however, the titer of full-length E1A, was 4 times lower in full fraction, indicating a higher degree of E1A fragmentation.

Because the dPCR measurements for intermediate fraction fell between the LOD and LOQ for assay 1, we aimed to evaluate whether the countable PCR platform with higher partitioning capacity and single-molecule counting could provide a more reliable estimation. For these reasons, the DNase I untreated intermediate fraction was further analyzed using countable PCR platform (Table 5). The resulting E1A measurements were within the expected range and indicated that 8.5% of molecules represented full-length fragments.

To further evaluate quantification accuracy and matrix effects, spike-recovery experiments were performed by adding defined amounts of synthetic gBlock DNA (ampicillin) or host cell DNA (E1A and SV40T) into the scAAV9 sample (Table 6). Due to the limited amount of AAV material, Bio-Rad dPCR analysis was performed using the full fraction, whereas countable PCR analysis was conducted using the intermediate fraction. Based on our previous study,[24] both fractions contained comparable amounts of full viral capsids and inputs were normalized accordingly. To determine percentage recovery measured targets' copy numbers in spiked samples were compared with theoretical values, calculated as the sum of the corresponding non-spiked AAV sample and spike-only control. For ampicillin, the original scAAV9 material contained high background levels of the ampicillin resistance gene. Therefore, AAV samples were first diluted 10,000-fold and contained approximately 1.3E+06 AAV capsids per reaction. Determined dPCR recoveries were 97%–100% across both duplex dPCR configurations, demonstrating accurate quantification. For E1A and SV40T, spike-recovery was evaluated using 1:10 and 1:100 dilutions of the AAV fraction (corresponding to approximately 1.3E+09 and 1.3E+08 AAV capsids per reaction respectively). At 1:10, recoveries were low (23.7%–71%) across spike levels and assay combinations, indicating strong matrix inhibition at high viral capsid loads. At 1:100, recoveries improved to 75.7%–137%. In contrast, the countable PCR platform showed lower inhibitory effect at 1:10 dilution of AAV sample for E1A spiked-ins experiments, enabling more accurate quantification even at high matrix load. Similarly to dPCR, no inhibition was observed at higher levels of AAV dilutions used for testing of recovery for ampicillin resistance gene in the countable PCR platform.

Orthogonal confirmation of DNA impurities via nanopore sequencing

Overall, nanopore sequencing of scAAV samples yielded datasets of consistent quality, with mean Q-scores ranging from 11.5 to 11.9 (Table S9). As expected, read length distributions were skewed toward shorter fragments, particularly in the heavy fraction; however, longer reads were also present (N50 ∼3.5–4.3 kb), supporting downstream analyses of genomic heterogeneity in the samples (Figure S9). In most fractions, the intended vector genome represented the major portion of reads (>80%). DNase I-treated samples, with exception of heavy fraction (H+), displayed a highly consistent mapping profile, with 92%–97% of reads aligning to the vector genome, 1%–3% to plasmid sequences, and 1%–5% to hcDNA. In contrast, the heavy DNase I treated (H+) and full untreated (F−) fractions exhibited a mixed composition, with approximately 80% of reads mapping to the vector genome, 3%–7% to plasmid DNA, and 15% to hcDNA. The heavy DNase I untreated fraction (H−) was dominated by hcDNA contamination (∼80%), with only 17% vector genome and 2% plasmid sequences detected. These findings indicate that hcDNA contamination is primarily associated with material outside AAV particles and concentrated in the highest-density regions of the CsCl ultracentrifugation gradient. In contrast, plasmid carryover remains minimal and is not significantly affected by DNase I pretreatment, suggesting that plasmid sequences are predominantly encapsidated within AAV particles.

We additionally examined the presence of our DNA impurity targets in the nanopore dataset. No specific mapping to SV40T and E1A

Table 4. Evaluation of ampicillin resistance gene in DNase I-treated and untreated-AAV9 fractions using digital PCR and countable PCR approach

			Amp											
			Titer (*N* of target/mL)						% Of target signal attributed to non-encapsidated DNA				% Linked molecules in multiplex	
Fraction	DNase I pretreatment	EGFP titer (*N* of target/mL)	Assay 1 (1 + 3)	Assay 2 (2 + 3)	Assay 3 (1 + 3)	Assay 3 (2 + 3)	Linked molecules (1 + 3)	Linked molecules (2 + 3)	1. (1 + 3)	3. (1 + 3)	2. (2 + 3)	3. (2 + 3)	1 + 3	2 + 3
Heavy	+	1.5E+11	1.6E+09	1.7E+09	1.7E+09	1.8E+09	1.1E+09	1.4E+09	93	93	93	92	54.6	71.7
	–	NA	2.2E+10	2.3E+10	2.3E+10	2.3E+10	1.6E+10	1.8E+10					58.9	69.0
Full	+	1.5E+12	2.9E+10	3.2E+10	4.3E+10	4.2E+10	1.9E+10	2.5E+10	31	24	25	22	39.4	55.1
	–	NA	4.3E+10	4.3E+10	5.6E+10	5.3E+10	2.5E+10	2.9E+10					37.6	47.7
Intermediate	+	1.5E+12	1.9E+10	2.2E+10	2.7E+10	2.7E+10	1.1E+10	1.3E+10	2	0	0	0	31.8	45.6
	–	NA	2.0E+10	2.1E+10	2.6E+10	2.6E+10	9.8E+09	1.3E+10					29.9	40.9
Intermediate[a]	–	NA	2.1E+10[a]	2.4E+10[a]	3.1E+10[a]		9.9E+09[a]		NA				22.9[a]	
Empty	+	2.4E+11	6.8E+09	6.6E+09	6.5E+09	6.4E+09	2.3E+09	2.7E+09	6	7	9	10	16.9	25.0
	–	NA	7.2E+09	7.3E+09	7.0E+09	7.1E+09	2.2E+09	2.9E+09					19.8	28.0

[a]Values determined in triplex countable PCR, where assays 1, 2, and 3 were used.Titers were determined separately for each developed assay, the proportion of non-encapsidated and unfragmented ampicillin gene was calculated using only dilutions above the LOQ. EGFP titers were previously determined and reported in Janc et al. (2024) and are included here for comparison. NA, not assessed in this study.

gene was observed. Initially, we used the original gBlock sequences for mapping and also attempted alignment with the adenovirus 5 genome and a larger region of the SV40T; however, no specific mapping was detected within the regions corresponding to our dPCR assay targets. We did, however, confirm the presence of the ampicillin resistance gene in all samples, although it represented only a small number of all mapped reads (Table 7; Figure S10). The heavy fraction contained the highest number of Amp resistance reads (2,906 treated; 4,292 untreated), while the full fraction showed 176 (treated) and 1,758 (untreated). Intermediate and empty fractions exhibited lower counts (204–500 and 285–336, respectively). Overall, these findings indicate that DNase I pretreatment substantially reduces extraneous DNA, particularly in heavy and full fractions, while plasmid-derived sequences remain detectable at low levels across all conditions. Additional analysis of reads mapping to ampicillin resistance gene revealed lower % of full-length ampicillin resistance gene as it was determined by our dPCR assays (Tables 4 and 7).

DISCUSSION

In this study, we developed and qualified several multiplex dPCR approaches capable of detecting and quantifying full-length and fragmented DNA impurities in rAAV preparations. By targeting terminal and internal regions of four relevant DNA impurities (ampicillin, kanamycin, and adenoviral E1A and SV40T gene), the assays enabled integrity evaluation at the sensitivity that conventional qPCR or next-generation sequencing approaches cannot achieve. The high linearity ($R^2 > 0.99$) and low limits of detection (9–22 copies/reaction) across all assays demonstrate the analytical robustness of the workflow and support its applicability to routine AAV vector characterization and process development. The observed % bias and absolute % bias were generally moderate (typically within ±20%) but increased in certain assay configurations and at low copy numbers. This variability likely reflects a combination of factors rather than a single source of error. As the input concentrations were based on a shared dilution series rather than independently quantified targets, part of the deviation may arise from assay-specific performance differences. In addition, the presence of fragmented template material, particularly affecting assays spanning distant or terminal regions, may lead to underestimation of copy numbers and contribute to the observed bias.

In addition to dPCR assay development, an important objective of this study was to evaluate whether the assays could be directly transferred to a countable PCR platform without major redesign. This would enable greater flexibility in platform selection while maintaining assay performance. Not all assay sets could be directly transferred to the countable PCR platform. Out of the four developed assay sets, only the Amp and E1A assays were successfully implemented without major modifications. These assays were therefore subjected to a more detailed evaluation of analytical performance, including linearity, LOD, LOQ, and LOB. Based on its substantially higher degree of partitioning and independence from Poisson-based quantification, countable PCR was expected to achieve lower LOD and LOQ values compared to dPCR. However, the observed

Table 5. Evaluation of SV40 large T antigen and E1A gene in DNase I untreated AAV9 fractions using digital PCR and countable PCR platform

	SV40T						E1A			
	Titer (*N* of target/mL)						Titer (*N* of target/mL)			
Fraction	Assay 1 (1 + 3)	Assay 2 (2 + 3)	Assay 3 (1 + 3)	Assay 3 (2 + 3)	Linked molecules (1 + 3)	% Full length impurities (1 + 3)	Assay 1 (1 + 2)	Assay 2 (1 + 2)	Linked molecules (1 + 2)	% Full length impurities (1 + 2)
Heavy	2.5E+05	<LOQ	1.2E+05	<LOQ	2.4E+04	7.2	5.8E+05	5.9E+05	2.1E+05	20.6
Full	1.3E+05	<LOQ	<LOQ	<LOQ	[b]	[b]	2.8E+05	3.0E+05	4.6E+04	9.3
Intermediate	<LOQ	<LOD	<LOD	<LOD	[b]	[b]	<LOQ	8.4E+04	[b]	[b]
Intermediate[a]	[c]						1.7E+05[a]	7.5E+04[a]	1.9E+04[a]	8.5[a]
Empty	<LOD	<LOD	<LOD	<LOD	[b]	[b]	<LOQ	7.2E+04	[b]	[b]

Titers were determined separately for each developed assay, and the proportion of unfragmented DNA impurities were calculated using only dilutions above the LOQ.
[a]Values determined in duplex countable PCR where assays 1 and 2 were used.
[b]Value could not be calculated because one or more assay titers were below the LOQ.
[c]Analysis not performed because the respective assay could not be implemented on the countable PCR platform, without further extensive optimization or re-design.

performance was comparable between the two platforms, with countable PCR even showing slightly higher LOD and LOQ values in certain cases. This apparent discrepancy suggests that increased partitioning alone does not directly translate into improved analytical sensitivity. Notably, a higher background signal was observed in NTC samples on the countable PCR platform, resulting in elevated LOB values. As LOD and LOQ are intrinsically dependent on background signals, this shift effectively increased the calculated detection limits, thereby offsetting the theoretical sensitivity advantage of increased partitioning.

As a technical observation, we noted that synthetic gBlocks used for assay development showed evidence of fragmentation (a phenomenon observed in our other projects as well), despite appearing predominantly full-length by size-based analysis using TapeStation capillary electrophoresis (Table 2; Figures S4 and S5). These results suggest that size-based electrophoretic methods may overestimate full-length integrity and that terminal degradation contributes to fragmentation in synthetic DNA standards. Notably, duplex assays targeting more closely spaced regions exhibited a higher fraction of double-positive partitions, indicating that shorter intact fragments are more likely to be detected when assay targets are in close proximity. In contrast, assays spanning more distant regions were more sensitive to fragmentation, particularly at the termini of the gBlock fragments. This effect is further amplified by assay design, as several targets are located close to fragment ends; consequently, both primer-probe sets depend on the integrity of terminal sequences, making these assays particularly susceptible to degradation-induced signal loss. While fragmentation was already evident in initial measurements, it should be noted that the gBlock material was subjected to multiple freeze-thaw cycles during extended testing, which may have further contributed to the observed fragmentation patterns. Application of the assays targeting host-cell DNA on genomic DNA extracted from the HEK293T cell line revealed clear differences in the abundance and integrity of the impurities in the tested DNA. Using dPCR, E1A was detected at 10-fold higher titers than SV40T and appeared approximately 90% full length compared to 60% determined for SV40T. These observations are consistent with genomic architecture of HEK293-derived cells, as the adenoviral E1A/E1B locus is present at 5–6 copies, while SV40 large T is present as a single integrated transgene in HEK 293T cell.[25]

The consistently lower percentage of full-length fragments detected by single-molecule counting using countable PCR, observed for both gBlocks and HEK293T-derived genomic DNA, is most likely attributable to methodological differences between the two platforms rather than to fundamental differences in the underlying fragment structure. Specifically, countable PCR enables single-molecule DNA counting through substantially higher numbers of partitions operating in a single-molecule occupancy regime, thereby increasing analytical resolution at low target concentrations. In addition, DNA integrity assessment by countable PCR was performed using a true triplex assay design for the ampicillin, allowing simultaneous detection of sequences at both ends and within the gene fragment in a single partition. This higher assay stringency increases sensitivity to partially fragmented molecules and is expected to yield lower apparent % integrity values. In contrast, integrity values derived from conventional dPCR were obtained using duplex reactions, which inherently rely on Poisson-derived linkage analysis between two spatially separated targets and therefore provides an approximate measure of full-length molecules that is inherently influenced by target spacing and actual co-localization of fragments in partitions. This difference in multiplexing strategy and molecular resolution likely explains the systematically higher % integrity values reported for dPCR.

For the E1A target, where duplex assay formats were used on both platforms, the observed differences in % integrity were substantially smaller and within the method variability (approximately, 5% for gBlocks and <10% for extracted genomic DNA), further supporting the conclusion that the larger discrepancies observed for antibiotic resistance targets arise primarily from presence of a third assay and platform-specific analytical resolution rather than biological differences.

Table 6. Percent recovery of spiked molecules (hcDNA or gBlock) containing targets in the AAV9 fractions using digital PCR and countable PCR

			Ampicillin				SV40T				E1A	
	Number of AAV capsids per reaction	Spiked level	Assay 1 (1 + 3)	Assay 2 (2 + 3)	Assay 3 (1 + 3)	Assay 3 (2 + 3)	Assay 1 (1 + 3)	Assay 2 (2 + 3)	Assay 3 (1 + 3)	Assay 3(2 + 3)	Assay 1 (1 + 2)	Assay 2 (1 + 2)
Digital PCR	1.3E+06	high	100	97	100	97	NA				NA	
	1.3E+09–1.5E+09	high	NA				47	39	30	29	36	35
		medium					53	40	30	24	43	55
		low					44	55	34	39	71	64
	1.3E+08–1.5E+08	high	NA				120	91	92	88	92	90
		medium					137	114	99	106	96	86
		low					105	105	76	99	94	96
Countable PCR[a]	2.6E+05	high	101[a]	98[a]	98[a]		b				NA	
	1.3E+09	high	NA								120	120
		medium									75	105
		low									67	111
	1.3E+08	high	NA								127	127
		medium									104	108
		low									93	95

NA, not assessed in this study.
[a]Values determined in triplex countable PCR, where assays 1, 2, and 3 were used.
[b]Analysis not performed because the respective assay could not be implemented on the countable PCR platform, without further extensive optimization or re-design.

The rAAV materials analyzed in this study were intentionally selected as impurity-enriched proof-of-concept samples to challenge assay sensitivity and assess DNA integrity under controlled conditions. Accordingly, the absolute levels of detected DNA impurities are not representative of clinical-grade AAV preparations but rather serve to validate the analytical performance of the workflow. Importantly, the methodology is directly transferable to highly purified AAV samples, where sensitive detection of low-abundance residual DNA may be even more critical. Analysis of tested CsCl-purified scAAV9 fractions revealed distinct impurity patterns between plasmid-derived and host cell-derived sequences. The ampicillin resistance gene was quantifiable across all fractions and remained abundant even after DNase I treatment, indicating that plasmid DNA was encapsidated within AAV particles (Table 4). This observation aligns with previous studies showing that AAV most frequently package backbone DNA elements, including antibiotic resistance genes, promoter fragments, and bacterial origins of replication.[14,19,20] Our results indicate that the full-length ampicillin resistance gene represents up to 1.3% of the total vector genome in DNase I-treated CsCl fractions. Notably, other studies using CsCl full fraction have reported comparable proportions of ampicillin resistance gene (targeting short amplicon) relative to total vector genome and also showed that those sequences can be transferred and persist *in vivo* after administration.[19] This proves that the backbone carryover is a reproducible phenomenon across rAAV preparations produced by transient transfection. It has been proven that plasmid backbone sequences get packed due to their close proximity to the Rep protein-binding sites (RBs) like ITRs on the pAAV-GOI plasmids or P5 promoter on the rep/cap plasmid.[14] Brimble and colleagues have also proven that P5 promoter-mediated packed sequences can also be translated and immunogenic, highlighting the need for accurate quantification of the full length of those sequences. Our dPCR analysis further demonstrated that the proportion of full-length ampicillin molecules varied across fractions, highest being in DNase I-untreated highest density fractions, where overfilled particles are expected, and lowest in the DNase I-treated empty fraction. In the full and intermediate fractions, ∼37% and ∼30% of encapsidated ampicillin fragments remained in full length. DNase I pretreatment had the strongest impact in the heavy fraction, reducing ampicillin signal by ∼90%. We hypothesize that longer plasmid fragments may associate with host-cell DNA or other macromolecular complexes, increasing particle density and promoting sedimentation into the heavy CsCl fraction. Not encapsidated DNA associated in this manner would be susceptible to nuclease treatment, consistent with pronounced DNase-dependent signal reduction observed in this fraction.

In contrast to the high titers of the ampicillin resistance gene across all fractions, host cell-derived DNA impurities (SV40T and E1A gene) were present at much lower levels, where DNase I treatment usually reduced their abundance below the LOD. We were able to quantify E1A and SV40T in the heavy and full DNase I untreated fractions (Table 5), where these targets most likely originate from extrinsic host cell DNA rather than from AAV capsids and can be therefore further removed in more advance purification protocols. Comparable observation has been reported for the lentiviral vector preparations produced in the HEK293T cell.[26] Although low levels of E1A and SV40 large transfer DNA were detectable using qPCR in purified

Table 7. Nanopore sequencing results including the total number of mapped reads, the number of reads mapped to all three plasmids, the number of reads mapped to the ampicillin resistance gene, and the percentage of reads covering both dPCR regions in the 1 + 3 and 2 + 3 duplex dPCR reactions

Fraction	DNase pretreatment	Mapped reads (count)	Recombinant plasmid reads (count)	AmpR reads (count)	Reads mapping to targets 1 & 3 (%)	Reads mapping to targets 2 & 3 (%)
Heavy	+	186,732	12,777	2,906	5.0	9.5
	–	639,475	13,756	4,292	33.6	43.8
Full	+	46,103	539	176	12.3	19.4
	–	276,238	10,392	1,758	4.9	8.3
Intermediate	+	34,827	563	204	13.2	19.0
	–	73,148	1,951	500	9.5	19.5
Empty	+	35,815	926	285	6.9	14.8
	–	44,668	1,197	336	7.1	14.2

material, subsequent experiments demonstrated that the passive transfer of those sequences to the target cell was minimal and the replication was not present, as the signal dropped under the LOD within one week.[26] This parallel underscores that small amounts of mostly fragmented helper-gene DNA can persist even after purification, yet exist at levels unlikely to pose functional risk, while simultaneously highlighting the value of sensitive quantitative assays for accurately profiling such residual impurities, most importantly their length. Spike-in experiments revealed a pronounced matrix-dependent inhibition effect in dPCR at high AAV concentrations, resulting in substantial underestimation of low-abundance targets at lower dilution levels, while increased dilution mitigated inhibition but reduced sensitivity and detection reliability. In contrast, countable PCR demonstrated greater robustness to matrix effects, maintaining accurate target recovery under high-background conditions, highlighting an inherent trade-off in dPCR between inhibition relief and sensitivity when quantifying low-level impurities in AAV samples.

Nanopore sequencing provided an orthogonal approach that confirmed high levels of plasmid derived impurities as well as high levels of host cell DNA. Vector genomes represented the majority of reads (>80%) in most fractions, while plasmid DNA appeared at low but consistent frequencies (1%–7%) (Figure S10). Similar results have been reported in another study using Nanopore sequencing.[12] Heavy untreated fractions contained substantial host-cell DNA, consistent with their expected enrichment in higher-density CsCl regions. Ampicillin reads were detected even after DNase I treatment, confirming encapsidation (Table 7). The lower proportion of full-length ampicillin reads detected by nanopore sequencing compared with dPCR full-length fragments likely reflects biases during library preparation. It has been shown for AAV vector genomes that, even when native, alkaline or capillary gel electrophoresis indicates presence of mainly full-length genomes, the percentage of full-length genomes determined by NGS can be significantly lower (e.g., ∼13% using PacBio sequencing).[11] No reads from our samples mapped to E1A or SV40T, consistent with their low abundance and high fragmentation. Sequencing depth was limited by low concentration of scAAV9 material, which required supplementation with lambda DNA; combined with the absence of second-strand synthesis, this may have reduced the detectability of rare or ssDNA impurities (e.g., vector-host cell chimera).

In clinically relevant AAV preparations, residual DNA is typically present at lower levels and predominantly in fragmented forms, particularly for host cell DNA, while plasmid-derived sequences are often detected as short backbone fragments. In contrast, our impurity-enriched CsCl fractions contained both fragmented host cell DNA and longer plasmid-derived sequences, consistent with known AAV packaging preferences favoring shorter DNA but permitting incorporation of longer replication-associated elements. These findings emphasize that, beyond quantification, assessing DNA integrity is critical for understanding potential biological impact and remains directly applicable to clinical-grade AAV samples.

In summary, this study introduced a practical and sensitive workflow for assessing DNA impurities in rAAV preparations. The multiplex dPCR approach provides high analytical sensitivity and enables discrimination between full-length and fragmented DNA molecules, representing a significant advancement in impurity characterization. Complementary analysis using countable PCR, achieved through straightforward adaptation of dPCR assays to UM chemistry and real-triplex detection, extended the dynamic range of quantification and enabled reliable detection of low-abundance targets below the LOQ of droplet-based duplex dPCR. Our findings confirm frequent packaging of plasmid backbone DNA into AAV capsids, while demonstrating that host-cell DNA is largely extrinsic and can be effectively reduced through nuclease treatment and improved purification.

Looking forward, further modifications in production may help reduce the biosafety risk of those impurities even more. Such strategies could include redesigning plasmid backbones to limit the packing of antibiotic resistance genes or engineering the production cell line to remove apparently non-essential elements like SV40T. Recent work indicates that CRISPR-Cas9-mediated deletion of SV40T from HEK293T cells does not significantly reduce the lentiviral vector or rAAV titers, although additional studies are needed to fully understand the broader impact of such modifications.[27] Together, these anticipated advances in production, combined with the development of

increasingly sensitive analytical tools, will enable tighter control over residual DNA impurities and contribute to the generation of safer rAAV products.

METHODS

Materials

gBlocks

To support initial testing of the newly developed assays, we designed four synthetic gBlocks: Amp, Kan, E1A, and SV40T. Since the SV40T genomic sequence was too long and prone to forming secondary structures, we truncated and modified regions outside the targeted areas to enable its synthesis and named this gBlock as "modified SV40T" (mSV40T). All genomic sequences were retrieved from NCBI database (accessed on August 20, 2022). The full-length sequences of gBlocks can be found in the supplemental information (Tables S2 and S3). The gBlocks were purchased from Integrated DNA Technologies (Coralville, IA, USA) and resuspended in 1× TE (echo time) buffer following manufacturer's instructions to reach final concentration of 10 ng/μL. To ensure that amplicons remain separated and accessible to primers and probes after restriction reaction, the UNAFold web server was used to evaluate potential dimerization and secondary structure formation.[28] The gBlocks were digested using different restriction enzymes (e.g., BaeGI, BstXI, AseI, Bpu10I, and SmaI) in different combinations to get fragments that include different targets (Figure 1). Details can be found in the supplemental information (Table S6). The reaction mixture contained 20 ng of each gBlock, 5–20 units of each enzyme, 1× NEBuffer r3.1 or 1× rCutSmart Buffer (New England Biolabs, NEB) and nuclease-free water (NFW). For the restriction control sample, NFW was added instead of the restriction enzyme. Those samples were later used as intact gBlock controls. Optimal buffers were determined using the NEBcloner website (New England Biolabs) and are detailed in the supplemental information (Table S6). All reaction mixtures were incubated at 37°C for 15 min and subsequently heat-inactivated at 80°C for 20 min, except for reactions containing SmaI, which were inactivated at 65°C for 20 min. Prior to dPCR, all restriction reactions were diluted in NFW supplemented with salmon sperm DNA at a final concentration of 2 ng/μL, to achieve the expected number of DNA targets per reaction.

We used the 4150 TapeStation automated electrophoresis system using Instrument Controller Software v.5.1.0.47235 with D5000 DNA ScreenTape (Agilent Technologies, Santa Clara, CA, USA) following manufacturer's instructions to determine the size and quantity of each fragment produced during the restriction reaction.

HEK293T cell genomic DNA

Genomic DNA from HEK293T cells was obtained from Cytion Biosciences (CLS Cell Lines Service GmbH, Eppelheim, Germany; ref. 388300189GD5).

AAV samples

Self-complementary AAV9 vectors expressing EGFP, produced via triple transfection in HEK293T cells and purified through two consecutive rounds of CsCl ultracentrifugation were used in the experiments. Detailed information on vector production, purification, and characterization are available in our previous publication.[24] During AAV material preparation, Benzonase (100 U/mL) and RNase (10 μg/mL) were added after PEG precipitation and prior to CsCl gradient ultracentrifugation, followed by incubation for 2 h at 37°C. For assay testing and qualification, we used four fractions: heavy (H) with a refractive index (RI) of 1.374, full (F) with an RI of 1.370, intermediate (I) with RI of 1.367 and empty (E) with RI of 1.364. Two technical replicates were combined to obtain enough material for dPCR testing and nanopore sequencing. Prior to dPCR testing and nanopore sequencing, fractions were treated with Ambion DNase I (Thermo Fisher Scientific, Waltham, MA, USA) to determine whether the detected impurities were encapsidated. Samples were also tested without DNase I treatment. To reach the dynamic range or to mitigate the inhibitory effects of DNase I during the dPCR, samples were further diluted 10-fold in a dPCR dilution buffer composed of PCR Buffer II without $MgCl_2$ (Applied Biosystems, Foster City, CA, USA), 25 mM $MgCl_2$ (Applied Biosystems), 0.05% Pluronic F-68 (Thermo Fisher Scientific), and sheared salmon sperm DNA (Thermo Fisher Scientific) at a final concentration of 2 ng/μL. Due to low levels of E1A gene and SV40T, samples were additionally tested without DNase I pretreatment and we only diluted 10 times in dPCR buffer prior dPCR.

For nanopore sequencing, DNase I was heat-inactivated with 0.5 M EDTA at 75°C for 10 min. The total DNA content of the fractions was extracted using the QIAamp MinElute Virus Spin Kit (QIAGEN, Hilden, Germany) following the manufacturer's protocol for purification of viral nucleic acids from plasma or serum, with minor modifications. DNA was eluted in 58 μL of NFW, and concentrations were determined using the Qubit dsDNA HS Assay Kit (Invitrogen, Carlsbad, CA, USA) according to the manufacturer's instructions. Because extracted DNA concentrations were low (≤10 ng/μL), samples were concentrated using 1.8× AMPure XP beads. After gentle mixing with pipette tips, samples were incubated for 15 min at room temperature on a hula mixer, followed by bead washing with 80% ethanol and resuspension in 13 μL of NFW. Since the total amount of DNA extracted from each fraction was lower than recommended (≤1 μg) for optimal library preparation using the ligation sequencing kit (SQK-LSK109), Lambda DNA control (part of the library preparation kit) previously digested with BstEII-HF was added to all samples prior library preparation. Libraries were prepared following the ligation sequencing gDNA Native Barcoding protocol (SQK-LSK109 with EXP-NBD104) available on the official Nanopore website. Barcoded and pooled libraries were loaded onto an R9.4.1 flow cell and sequenced for 72 h.

Methods

dPCR assay development and optimization

For each of the targets, we have developed 2 or 3 different assays to detect distinct fragments (one at the beginning, one in the middle, and one at the end of each targeted-DNA impurity (Figure 1; Tables S1–S3) to determine the full length of these impurities. Assays were developed using Primer Express 2.0 software (Thermo Fisher Scientific, USA) as described previously.[29]

dPCR reaction conditions

Assays were first evaluated using intact gBlocks, followed fragmented gBlocks, and finally on AAV samples. All dPCR experiments were performed on the QX200 Droplet Digital PCR platform (Bio-Rad, USA). Four independent serial dilutions of intact gBlocks were prepared, each containing eight dilution points (80,000; 10,000; 2,000; 1,000; 100; 50; 20; and 10 targets per reaction). Two duplex configurations were tested: assays 1 and 2, both labeled with FAM dye, were used interchangeably and paired with assay 3, labeled with HEX dye.

The 15 μL dPCR reaction mixture contained 10 μL of 2× ddPCR Supermix for Probes (no dUTP), 1 μL of each assay (either assay 1 or assay 2 and assay 3) primer/probe mix, and NFW added to a final volume of 15 μL. The reaction mixture was briefly vortexed and transferred to 96-well plate (Eppendorf, Germany), where 5 μL of DNA (gBlock dilution, fragmented gBlocks, HEK293T cell genomic DNA or AAV sample) was added. All dilution points were tested in duplicate. Droplets were generated using the QX200 Automated Droplet Generator (Bio-Rad). The PCR cycling protocol was 10 min at 95°C followed by 40 cycles of 30 s at 95°C and 1 min at 60°C (ramp rate of 2.5°C/s), followed by 98°C for 10 min. After cycling, the plate was transferred to the QX200 Droplet Reader (Bio-Rad) and results were initially processed using QX Manager software v.2.2.0 (Bio-Rad).

Thresholds for positive/negative droplets discrimination were set manually using one-dimensional amplitude plots and refined using two-dimensional plots when needed. Raw output data, including the number of positive and accepted droplets, as well as calculated linked-molecule concentration, were exported and further processed in Microsoft Excel. Results obtained from intact gBlocks were used to determine the LOD, LOQ, and linearities of the assays. LOD was defined as the minimum copy number concentration that can be reliably detected, meaning all technical replicates are positive (≥3 positive droplets).[30] LOQ was defined as the copy number concentration at which the sample can be reliably quantified (i.e., that the coefficient of variation (CV) of all technical replicates for that dilution is below 20%, and all higher dilution points also have CV values below 20%) (Table S4). Additionally, the % bias between expected and measured copies per reaction at each dilution level was calculated as (Equation 1):

$$\% \, Bias = \frac{mean \ measured \ copies \ per \ reaction - expected \ copies \ per \ reaction}{expected \ copies \ per \ reaction} \times 100, \quad \text{(Equation 1)}$$

where the mean measured values were calculated from replicate measurements used in the linearity assessment. Negative values indicate underestimation, whereas positive values indicate overestimation. Additionally, % bias was also reported as absolute values.

Countable PCR assays development and reaction conditions

Hydrolysis probe assays developed for dPCR workflow were converted to Countable Lab's (CL) UM chemistry by appending universal sequences to the original target-specific primers (IDT). This allowed detection using standardized fluorescent probes. The countable PCR workflow supported true triplex reactions for SV40T, ampicillin, and kanamycin, whereas the E1A assays stayed in the duplex configuration. Developed assays were tested on the same DNA inputs as for dPCR (gBlocks for initial testing and determination of LOD and LOQ, HEK 293T genomic DNA, and intermediate scAAV9 fraction). Each 50 μL reaction processed using the countable PCR workflow ([C]), which partitions samples into ∼30 million compartments via centrifugation to achieve single-molecule isolation and amplification. Thermal cycling consisted of 95°C (5 min) followed by 40 cycles of 95°C (20 s), 60°C (30 s), and 68°C (30 s). Following light-sheet fluorescence imaging, direct molecule counts were obtained using Countable Control Software (v.2.0). Linkage analysis was applied to determine DNA impurity integrity. All countable PCR workflows are performed according to the manufacturer's protocol. The limit of blank (LOB), LOD, and LOQ for countable PCR were determined according to the following criteria (Table S4):

The LOB was calculated using Equation 2:

$$LOB = mean \ of \ blank \ reactions + 1.645 \times SD \ of \ blank \ reactions. \quad \text{(Equation 2)}$$

The LOD was calculated using Equation 3:

$$LOD = LOB + 1.645 \times SD \ of \ reactions \ targeting \ 10 \ counts \ per \ reactions. \quad \text{(Equation 3)}$$

The LOQ was defined similarly to the dPCR assay as the mean counts per reaction at the lowest target concentration with a %CV below or equal 20% across technical replicates.

Spike-in experiments for assay performance characterization

Spike-in experiments were performed to assess the % recovery for both dPCR and countable PCR. Measured copy numbers in spiked samples were compared with theoretical values calculated as the sum of the non-spiked AAV sample and spike-only control (Equation 4):

$$\% \, PCR \ recovery = \frac{mean \ measured \ copies \ (or \ conts) \ per \ reaction \ in \ AAV \ spiked \ samples}{theoretical \ SUM \ of \ copies \ (or \ counts) \ per \ reaction \ of \ AAV \ only \ AND \ spike \ only \ samples} \times 100. \quad \text{(Equation 4)}$$

For ampicillin, the scAAV9 material contained a high endogenous background of ampicillin resistance gene sequences. Therefore, AAV samples were pre-diluted 10,000-fold (dPCR) and 100,000-fold (countable PCR) prior to spiking to ensure measurements were within the dynamic range. At this dilution, reactions contained approximately 1.3E+06 (dPCR) and 2.6E+05 (countable PCR) AAV capsids per reaction. Synthetic ampicillin gBlock DNA was then added to generate the defined spike level (approximately 12,000 targets/reaction). Corresponding AAV-only controls (water added instead of spiked gBlock) and gBlock-only controls (AAV matrix replaced with water) were prepared and analyzed in parallel.

For E1A and SV40T, the original scAAV9 material contained low amounts of targets and was therefore less diluted than in the case of ampicillin. AAV sample was diluted only 10 and 100 times (for both dPCR and E1A countable PCR). At 1:10 and 1:100 dilutions, each reaction contained approximately 1.3–1.5E+09 or 1.3–1.5E+08 AAV capsids, respectively. HEK293T genomic DNA was then added to generate high (200 copies/reaction), medium (100 copies/reaction), and low (60 copies/reaction) spike levels. Corresponding AAV-only and spike-only controls were prepared in parallel.

Nanopore sequencing analysis

Raw FAST5 reads were converted into POD5 format using Pod5 software (v.0.3.15). Dorado (v.0.7.2+9ac85c6) was then used for basecalling and demultiplexing of all reads. The initial length and quality of debarcoded reads were assessed using NanoStat (v.1.6.0) and NanoQC (v.0.9.4). Library adapters were trimmed and reads shorter than 50 bp were filtered using NanoFilt (v.2.8.0). Read mapping was performed with Minimap2 (v.2.26-r1175). SAM files were imported into CLC Genomics Workbench (v.25.0.2, QIAGEN), where mapping quality, average read length, and coverage were analyzed. Unmapped reads were filtered, exported, and remapped to subsequent references. General mapping included Lambda DNA, vector genome, pITR, pHelper, pAAV2/9, and host cell DNA (hg38 reference). For detailed analysis of DNA impurities (ampicillin resistance gene, SV40T, and E1A gene), all Lambda DNA unmapped reads were mapped to reference sequences of these impurities. Since no specific mapping was observed for E1A and SV40T, reads were additionally mapped to the AdV5 genome and the broader SV40T region. Reads mapped to the ampicillin resistance gene were analyzed using CLC Genomics Workbench. BED files containing the start and end positions of each read were exported and further processed in Excel. Based on their start and end positions, reads were grouped and classified into categories; reads covering only one target region (target 1, 2, or 3) and reads spanning over both dPCR target sites.

DATA AND CODE AVAILABILITY

Data is available on request from the authors. Raw sequencing data has been deposited in the European Nucleotide Archive (ENA) under study accession number PRJEB108885.

ACKNOWLEDGMENTS

The authors would like to thank Nika Savodnik for contribution to initial assay design, Tjaša Jakomin for the initial testing of the dPCR assays, and Lorena Zentilin from ICGEB for providing the AAV9 material. The authors would like to thank Emily Blair and Michael Balamotis from Countable Labs for converting and testing the assays on the countable PCR platform.

The authors acknowledge the Slovenian Research Agency (ARIS) for supporting research infrastructure used in this work through grant IO-0004 and the Slovenian node of Metrofood ERIC for providing access to dPCR. The work described in this manuscript was funded by the Slovenian Research and Innovation Agency (research core funding P4-0463, project L4-3180, 1000-20-0105, 1000-21-0105, and S-ZDR/22-27/0105).

The graphical abstract was created with BioRender.com (https://app.biorender.com/[accessed on September 21, 2026]). Figures 1, S4, S6, and S7 were created in Microsoft PowerPoint Presentation and exported in TIFF format.

AUTHOR CONTRIBUTIONS

M.J. and D.D contributed to the conceptualization of the study, developed methodology, data curation, and formal analysis; M.J. wrote the original draft; D.D. provided supervision; M.J. and D.D. contributed to reviewing and editing of the manuscript.

DECLARATION OF INTERESTS

D.D. acts as chief scientific officer at Niba Labs d.o.o., a company providing analytical development services to gene therapy developers.

DECLARATION OF GENERATIVE AI AND AI-ASSISTED TECHNOLOGIES IN THE MANUSCRIPT PREPARATION PROCESS

During the preparation of this manuscript the author, M.J. used an AI-based tool (Microsoft Copilot) solely for minor language editing to improve the readability of the manuscript. Both the authors reviewed and edited all the content and took full responsibility for the scientific accuracy and integrity of the manuscript.

SUPPLEMENTAL INFORMATION

Supplemental information can be found online at https://doi.org/10.1016/j.omta.2026.201854.

REFERENCES

1. Lukashev, A.N., and Zamyatnin, A.A. (2016). Viral vectors for gene therapy: Current state and clinical perspectives. Biochemistry. *81*, 700–708. https://doi.org/10.1134/S0006297916070063.
2. Wang, J.-H., Gessler, D.J., Zhan, W., Gallagher, T.L., and Gao, G. (2024). Adeno-associated virus as a delivery vector for gene therapy of human diseases. Signal Transduct. Targeted Ther. *9*, 78. https://doi.org/10.1038/s41392-024-01780-w.
3. Wilmott, P., Lisowski, L., Alexander, I.E., and Logan, G.J. (2019). A User's Guide to the Inverted Terminal Repeats of Adeno-Associated Virus. Hum. Gene Ther. Methods *30*, 206–213. https://doi.org/10.1089/hgtb.2019.276.
4. Park, M.T., Verma, A., Froelich, C.A., and Motevalian, S.P. (2025). Process and quality considerations for recombinant adeno-associated virus manufacturing platforms. Trends Biotechnol. *43*, 1921–1937. https://doi.org/10.1016/j.tibtech.2025.02.016.
5. Tan, E., Chin, C.S.H., Lim, Z.F.S., and Ng, S.K. (2021). HEK293 Cell Line as a Platform to Produce Recombinant Proteins and Viral Vectors. Front. Bioeng. Biotechnol. *9*, 796991. https://doi.org/10.3389/fbioe.2021.796991.
6. Meier, A.F., Fraefel, C., and Seyffert, M. (2020). The Interplay between Adeno-Associated Virus and Its Helper Viruses. Viruses *12*, 662. https://doi.org/10.3390/v12060662.
7. Singhal, G., Leo, E., Setty, S.K.G., Pommier, Y., and Thimmapaya, B. (2013). Adenovirus E1A Oncogene Induces Rereplication of Cellular DNA and Alters DNA Replication Dynamics. J. Virol. *87*, 8767–8778. https://doi.org/10.1128/JVI.00879-13.

8. Ahuja, D., Sáenz-Robles, M.T., and Pipas, J.M. (2005). SV40 large T antigen targets multiple cellular pathways to elicit cellular transformation. Oncogene *24*, 7729–7745. https://doi.org/10.1038/sj.onc.1209046.

9. Brimble, M.A., Winston, S.M., and Davidoff, A.M. (2023). Stowaways in the cargo: Contaminating nucleic acids in rAAV preparations for gene therapy. Mol. Ther. *31*, 2826–2838. https://doi.org/10.1016/j.ymthe.2023.07.025.

10. Tran, N.T., Heiner, C., Weber, K., Weiand, M., Wilmot, D., Xie, J., Wang, D., Brown, A., Manokaran, S., Su, Q., et al. (2020). AAV-Genome Population Sequencing of Vectors Packaging CRISPR Components Reveals Design-Influenced Heterogeneity. Mol. Ther. Methods Clin. Dev. *18*, 639–651. https://doi.org/10.1016/j.omtm.2020.07.007.

11. Tran, N.T., Lecomte, E., Saleun, S., Namkung, S., Robin, C., Weber, K., Devine, E., Blouin, V., Adjali, O., Ayuso, E., et al. (2022). Human and Insect Cell-Produced Recombinant Adeno-Associated Viruses Show Differences in Genome Heterogeneity. Hum. Gene Ther. *33*, 371–388. https://doi.org/10.1089/hum.2022.050.

12. Dunker-Seidler, F., Breunig, K., Haubner, M., Sonntag, F., Hörer, M., and Feiner, R.C. (2025). Recombinant AAV batch profiling by nanopore sequencing elucidates product-related DNA impurities and vector genome length distribution. Mol. Ther. Methods Clin. Dev. *33*, 101417. https://doi.org/10.1016/j.omtm.2025.101417.

13. Tai, P.W.L., Xie, J., Fong, K., Seetin, M., Heiner, C., Su, Q., Weiand, M., Wilmot, D., Zapp, M.L., and Gao, G. (2018). Adeno-associated Virus Genome Population Sequencing Achieves Full Vector Genome Resolution and Reveals Human-Vector Chimeras. Mol. Ther. Methods Clin. Dev. *9*, 130–141. https://doi.org/10.1016/j.omtm.2018.02.002.

14. Brimble, M.A., Cheng, P.-H., Winston, S.M., Reeves, I.L., Souquette, A., Spence, Y., Zhou, J., Wang, Y.-D., Morton, C.L., Valentine, M., et al. (2022). Preventing packaging of translatable P5-associated DNA contaminants in recombinant AAV vector preps. Mol. Ther. Methods Clin. Dev. *24*, 280–291. https://doi.org/10.1016/j.omtm.2022.01.008.

15. Wright, J.F. (2014). Product-Related Impurities in Clinical-Grade Recombinant AAV Vectors: Characterization and Risk Assessment. Biomedicines *2*, 80–97. https://doi.org/10.3390/biomedicines2010080.

16. Higashiyama, K., Yuan, Y., Hashiba, N., Masumi-Koizumi, K., Yusa, K., and Uchida, K. (2023). Quantitation of Residual Host Cell DNA in Recombinant Adeno-Associated Virus Using Droplet Digital Polymerase Chain Reaction. Hum. Gene Ther. *34*, 578–585. https://doi.org/10.1089/hum.2023.006.

17. De, B.P., Cram, S., Lee, H., Rosenberg, J.B., Sondhi, D., Crystal, R.G., and Kaminsky, S.M. (2023). Assessment of Residual Full-Length SV40 Large T Antigen in Clinical-Grade Adeno-Associated Virus Vectors Produced in 293T Cells. Hum. Gene Ther. *34*, 697–704. https://doi.org/10.1089/hum.2023.032.

18. Wang, Y., Cooper, R., Kiladjian, A., Bergelson, S., and Feschenko, M. (2019). A Digestion-free Method for Quantification of Residual Host Cell DNA in rAAV Gene Therapy Products. Mol. Ther. Methods Clin. Dev. *13*, 526–531. https://doi.org/10.1016/j.omtm.2019.05.005.

19. Chadeuf, G., Ciron, C., Moullier, P., and Salvetti, A. (2005). Evidence for Encapsidation of Prokaryotic Sequences during Recombinant Adeno-Associated Virus Production and Their in Vivo Persistence after Vector Delivery. Mol. Ther. *12*, 744–753. https://doi.org/10.1016/j.ymthe.2005.06.003.

20. Jen, H.-I., Wilkinson, P., Lu, X., and Zhang, W. (2025). Residual DNA impurities in AAV vectors—nature and transcription. Mol. Ther. Methods Clin. Dev. *33*, 101503. https://doi.org/10.1016/j.omtm.2025.101503.

21. Buddle, S., Brown, L.-A.K., Morfopoulou, S., Torres Montaguth, O.E., Scoto, M., Herder, V., Dhawan, A., Brown, J.R., Atkinson, L., Kopec, A., et al. (2026). Contaminating plasmid sequences and disrupted vector genomes in the liver following adeno-associated virus gene therapy. Nat. Med. *32*, 472–480. https://doi.org/10.1038/s41591-025-04073-z.

22. Shum, E.Y., Lai, J.H., Li, S., Lee, H.G., Soliman, J., Raol, V.K., Lee, C.K., Fodor, S.P.A., and Fan, H.C. (2022). Next-Generation Digital Polymerase Chain Reaction: High-Dynamic-Range Single-Molecule DNA Counting via Ultrapartitioning. Anal. Chem. *94*, 17868–17876. https://doi.org/10.1021/acs.analchem.2c03649.

23. Lai, J.H., Keum, J.W., Lee, H.G., Molaei, M., Blair, E.J., Li, S., Soliman, J.W., Raol, V.K., Barker, C.L., Fodor, S.P.A., et al. (2023). New realm of precision multiplexing enabled by massively-parallel single molecule UltraPCR. Preprint at bioRxiv. https://doi.org/10.1101/2023.10.09.561546.

24. Janc, M., Zevnik, K., Dolinar, A., Jakomin, T., Štalekar, M., Bačnik, K., Kutnjak, D., Žnidarič, M.T., Zentilin, L., Fedorov, D., and Dobnik, D. (2024). In-Depth Comparison of Adeno-Associated Virus Containing Fractions after CsCl Ultracentrifugation Gradient Separation. Viruses *16*, 1235. https://doi.org/10.3390/v16081235.

25. Lin, Y.-C., Boone, M., Meuris, L., Lemmens, I., Van Roy, N., Soete, A., Reumers, J., Moisse, M., Plaisance, S., Drmanac, R., et al. (2014). Genome dynamics of the human embryonic kidney 293 lineage in response to cell biology manipulations. Nat. Commun. *5*, 4767. https://doi.org/10.1038/ncomms5767.

26. Merten, O.W., Charrier, S., Laroudie, N., Fauchille, S., Dugué, C., Jenny, C., Audit, M., Zanta-Boussif, M.-A., Chautard, H., Radrizzani, M., et al. (2011). Large-Scale Manufacture and Characterization of a Lentiviral Vector Produced for Clinical Ex Vivo Gene Therapy Application. Hum. Gene Ther. *22*, 343–356. https://doi.org/10.1089/hum.2010.060.

27. Bae, D.H., Marino, M., Iaffaldano, B., Fenstermaker, S., Afione, S., Argaw, T., McCright, J., Kwilas, A., Chiorini, J.A., Timmons, A.E., and Reiser, J. (2020). Design and Testing of Vector-Producing HEK293T Cells Bearing a Genomic Deletion of the SV40 T Antigen Coding Region. Mol. Ther. Methods Clin. Dev. *18*, 631–638. https://doi.org/10.1016/j.omtm.2020.07.006.

28. Zuker, M. (2003). Mfold web server for nucleic acid folding and hybridization prediction. Nucleic Acids Res. *31*, 3406–3415. https://doi.org/10.1093/nar/gkg595.

29. Dobnik, D., Kogovšek, P., Jakomin, T., Košir, N., Tušek Žnidarič, M., Leskovec, M., Kaminsky, S.M., Mostrom, J., Lee, H., and Ravnikar, M. (2019). Accurate quantification and characterization of adeno-associated viral vectors. Front. Microbiol. *10*, 1570. https://doi.org/10.3389/fmicb.2019.01570.

30. Bogožalec Košir, A., Muller, S., Žel, J., Milavec, M., Mallory, A.C., and Dobnik, D. (2023). Fast and Accurate Multiplex Identification and Quantification of Seven Genetically Modified Soybean Lines Using Six-Color Digital PCR. Foods *12*, 4156. https://doi.org/10.3390/foods12224156.

OMTA, Volume *34*

Supplemental information

Development and qualification of quantitative assays for detecting process-related nucleic acid impurities in recombinant AAV preparations

Mojca Janc and David Dobnik

Table S1: Digital PCR primers and probes developed and used in this study. Forward and reverse primers were used at the final concentration of 900 nmol/L and probes at 250 nmol/L.

DNA impurity target	Assay number	Assay coordinates on gBlock (bp)	Label	DNA Sequence of oligonucleotide (5' to 3')
**Amp**	1	20–82	Amp_res1 FP	GTGTCGCCCTTATTCCCTTTT
			Amp_res1 RP	GCGTTTCTGGGTGAGCAAA
			Amp_res1 Probe	FAM-TGCGGCATT/ZEN/TTGCCTTCC -BkFQ
	2	219–280	Amp_res2 FP	GCTATGTGGCGCGGTATTATC
			Amp_res2 RP	GTATGCGGCGACCGAGTT
			Amp_res2 Probe	FAM-CGTATTGAC/ZEN/GCCGGGCAAGAG -BkFQ
	3	676–745	Amp_res3 FP	GGCTGGTTTATTGCTGATAAATCT
			Amp_res3 RP	GCCCCAGTGCTGCAATG
			Amp_res3 Probe	HEX-CCGGTGAGC/ZEN/GTGGGTCTCGC -BkFQ
**Kan**	1	75–150	Kan_res1 FP	AAAGCCGTTTCTGTAATGAAGGA
			Kan_res1 RP	GCAGACCGATACCAGGATCTTG
			Kan_res1 Probe	FAM-AAAACTCAC/ZEN/CGAGGCAGTTCCATAGGATG-BkFQ
	2	245–309	Kan_res2 FP	TCCGGTGAGAATGGCAAAA
			Kan_res2 RP	CGTAATGGCTGGCCTGTTG
			Kan_res2 Probe	FAM-CTTATGCAT/ZEN/TTCTTTCCAGACTTG-BkFQ
	3	672–743	Kan_res3 FP	CATCGGGCTTCCCATACAAT
			Kan_res3 RP	GGTATAAATGGGCTCGCGATAA
			Kan_res3 Probe	HEX-TAGATTGTC/ZEN/GCACCTGATTGCCCGA-BkFQ
**SV40T**	1	119–191	LT-ant 1 FP	GCCTGTTTCATGCCCTGAGT
			LT-ant 1 RP	CAGCCAGGAAAATGCTGATAAA
			LT-ant 1 Probe	FAM-TCCATGTTC/ZEN/TTCTCCCCACCATCTTCA-BkFQ
	2	1812–1895	LT-ant 2 FP	TCCAATTATGTCACACCACAGAAGT
			LT-ant 2 RP	GCTACTGCTTCGATTGCTTTAGAA
			LT-ant 2 Probe	FAM-AGGTTCCTT/ZEN/CACAAAGATCAAGTCCAAACCA-BkFQ
	3	2190–2274	LT-ant 3 FP	AAATATGCCTTTCTCATCAGAGGAA
			LT-ant 3 RP	GAGAGGAATCTTTGCAGCTAATGG
			LT-ant 3 Probe	HEX-TCCCCCAGG/ZEN/CACTCCTTTCAAGACCTA-BkFQ
**E1A**	1	45–113	E1a 1 FP	GGCCGCCAGTCTTTTGG
			E1a 1 RP	AAATGGCTAGGAGGTGGAAGATT
			E1a 1 Probe	FAM-CCAGCTGAT/ZEN/CGAAGAGGTACTGGCTGA-BkFQ
	2	874–940	E1a 2 FP	AACCAGTTGCCGTGAGAGTT
			E1a 2 RP	TCGTTAAGCAAGTCCTCGATACAT
			E1a 2 Probe	HEX-TGGGCGTCG/ZEN/CCAGGCTGTG-BkFQ

Table S2: Full-length DNA sequences of plasmid derived gBlocks with marked dPCR assay coordinates. All assay coordinates are shown in bold; additionally, assay 2 is double-underlined and assay 3 is dashed-underlined.

gBlock name	DNA impurity target	gBlock length (bp)	Assay	Assay start and end position	gBlock sequence (5' to 3')
**Amp**	Ampicillin resistance gene	861	1<br>2<br>3	**20–82**<br>**219–280**<br>**676–745**	1 ATGAGTATTCAACATTTCC**GTGTCGCCCTTATTCCCTTTTTTGCGGCATT**<br>51 **TTGCCTTCCTGTTTTTGCTCACCCAGAAACGC**TGGTGAAAGTAAAAGATG<br>101 CTGAAGATCAGTTGGGTGCACGAGTGGGTTACATCGAACTGGATCTCAAC<br>151 AGCGGTAAGATCCTTGAGAGTTTTCGCCCCGAAGAACGTTTTCCAATGAT<br>201 GAGCACTTTTAAAGTTCT**GCTATGTGGCGCGGTATTATCCCGTATTGACG**<br>251 **CCGGGCAAGAGCAACTCGGTCGCCGCATAC**ACTATTCTCAGAATGACTTG<br>301 GTTGAGTACTCACCAGTCACAGAAAAGCATCTTACGGATGGCATGACAGT<br>351 AAGAGAATTATGCAGTGCTGCCATAACCATGAGTGATAACACTGCGGCCA<br>401 ACTTACTTCTGACAACGATCGGAGGACCGAAGGAGCTAACCGCTTTTTTG<br>451 CACAACATGGGGGATCATGTAACTCGCCTTGATCGTTGGGAACCGGAGCT<br>501 GAATGAAGCCATACCAAACGACGAGCGTGACACCACGATGCCTGTAGCAA<br>551 TGGCAACAACGTTGCGCAAACTATTAACTGGCGAACTACTTACTCTAGCT<br>601 TCCCGGCAACAATTAATAGACTGGATGGAGGCGGATAAAGTTGCAGGACC<br>651 ACTTCTGCGCTCGGCCCTTCCGGCT**GGCTGGTTTATTGCTGATAAATCTG**<br>701**GAGCCGGTGAGCGTGGGTCTCGCGGTATCATTGCAGCACTGGGGC**CAGAT<br>751 GGTAAGCCCTCCCGTATCGTAGTTATCTACACGACGGGGAGTCAGGCAAC<br>801 TATGGATGAACGAAATAGACAGATCGCTGAGATAGGTGCCTCACTGATTA<br>851 AGCATTGGTAA
**Kan**	Kanamycin resistance gene	816	1<br>2<br>3	**75–150**<br>**245–309**<br>**672–743**	1 TTAGAAAAACTCATCGAGCATCAAATGAAACTGCAATTTATTCATATCAG<br>51 GATTATCAATACCATATTTTTGAA**AAAGCCGTTTCTGTAATGAAGGAGAA**<br>101 **AACTCACCGAGGCAGTTCCATAGGATGGCAAGATCCTGGTATCGGTCTGC**<br>151 GATTCCGACTCGTCCAACATCAATACAACCTATTAATTTCCCCTCGTCAA<br>201 AAATAAGGTTATCAAGTGAGAAATCACCATGAGTGACGACTGAA**TCCGGT**<br>251 **GAGAATGGCAAAAGTTTATGCATTTCTTTCCAGACTTGTTCAACAGGCCA**<br>301 **GCCATTACG**CTCGTCATCAAAATCACTCGCATCAACCAAACCGTTATTCA<br>351 TTCGTGATTGCGCCTGAGCGAGACGAAATACGCGATCGCTGTTAAAAGGA<br>401 CAATTACAAACAGGAATCGAATGCAACCGGCGCAGGAACACTGCCAGCGC<br>451 ATCAACAATATTTTCACCTGAATCAGGATATTCTTCTAATACCTGGAATG<br>501 CTGTTTTCCCAGGGATCGCAGTGGTGAGTAACCATGCATCATCAGGAGTA<br>551 CGGATAAAATGCTTGATGGTCGGAAGAGGCATAAATTCCGTCAGCCAGTT<br>601 TAGTCTGACCATCTCATCTGTAACATCATTGGCAACGCTACCTTTGCCAT<br>651 GTTTCAGAAACAACTCTGGCG**CATCGGGCTTCCCATACAATCGATAGATT**<br>701 **GTCGCACCTGATTGCCCGACATTATCGCGAGCCCATTTATACC**CATATAA<br>751 ATCAGCATCCATGTTGGAATTTAATCGCGGCCTAGAGCAAGACGTTTCCC<br>801 GTTGAATATGGCTCAT

Table S3: Full-length DNA sequences of gBlocks derived from host cell DNA impurities with marked dPCR assay coordinates. All assay coordinates are shown in bold; additionally, assay 2 is double-underlined and assay 3 is dashed-underlined.

gBlock name	DNA impurity target	gBlock length (bp)	Assay	Assay start and end position	gBlock sequence (5' to 3')
**mSV40T**	SV40 large T antigen	2293			1 TTATGTTTCAGGTTCAGAAGCAAGTAAAACCTCTACAAATGTGGTATGGC
					51 TGATTATGATCATGAACAGACTGTGAGGACTGAGGGGCCTGAAATGAGCC
					101 TTGGGACTGTGAATCAAT**GCCTGTTTCATGCCCTGAGTCTTCCATGTTCT**
					151 **TCTCCCCACCATCTTCATTTTTATCAGCATTTTCCTGGCTG**TCTTCATCA
					201 TCATCATCACTGTTTCTTAGCCAATCTAAAACTCCAATTCCCATAGCCAC
					251 ATTAAACTTCATTTTTTGATACACTGACAAACTAAACTCTTTGTCCAATC
					301 TCTCTTTCCACTCCACAATTCTGCTCTGAATACTTTGAGCAAACTCAGCC
			1	**119–191**	351 ACAGGTCTGTACCAAATTAACATAAGAAGCAAAGCAATGCCACTTTGAAT
					401 AACAAAAACTCACTGCGTTCCAGGCAATGCTTTAAATAATCTTTGGGCCT
					451 AAACAAATCTGGCCTGCAGTCACACTGTACTCATTCATGGTGACTATTCC
					501 AGGGGGAAATATTTGAGTTACCTTAACACTGCCATCCAAATAATCCCTTA
					551 AATTGTCCAGGTTATTAATTCCCTGACCTGAAGGCAAATCTCTGGACTCC
					601 CCTCCAGTGCCCTTTACATCCTCAAAAACTACTAAAAACTGGTCAATAGC
					651 TACTCCTAGCTCAAAGTTCAGCCTGTCCAAGGGCAAATTAACATTTAAAG
					701 CTTTCCCCCCACATAATTCAAGCAAAGCAGCTGCTAATGTAGTTTTACCA
					751 CTATCAATTGGTCCTTTAAACAGCCAGTAGGAATGTTGTACACCATGCAT
					801 TTTAAAAAGTCATACACCACTGAATCCATTTTGGGCAACAAACAGTGTAG
					851 CCAAGCAACTCCAGCCATCCATTCTTCTATGTCAGCAGAGCCTGTAGAAC
					901 CAAACATTATATCCATCCTATCCAAAAGATCATTAAATCTGTTTGTTAAC
					951 ATTTGTTCTCTAGTTAATTGTAGGCTATCAACCCGTAAAACAGTATCAAC
					1001 AGCCTGTTGGCATATGCTGTCAGCAAATATAGCAGCATTTGCATAATGCT
					1051 TTTCATGGTACTTATAGTGGCTGGGCTGTATACATTTTAAACACATTTCA
					1101 AAACTGTACTGAAATTCCAAGTACATCCCAAGCAATAACAACACATCATC
			2	**1812–1895**	1151 ACATTGCATACTCTGTTACAAGCTTCCAGGACACTTGTTTAGTTTCCTCT
					1201 GCTTCTTCTGGATTAAAATCATGCTCCTTTAACCCACCTGGCAAACTTTC
					1251 CTCAATAACAGAAAATGGATCTCTAGTCAAGGCACTATACATCAAATATT
					1301 CCTTATTAACCCCTTTACAAATTAAAAAGCTAAAGGTACACAATTTTTGA
					1351 GCATAGTTATTAATAGCAGACACTCTATGCCTGTGTGGAGTAAAGTATGT
					1401 TATGATTATAACTGTTATGCCTACTTATAAAGGTTACAGAATATTTTTCC
					1451 ATAATTTTCTTGTATAGCAGTGCAGCTTTTTCCTTTGTGGTGTAAATAGC
					1501 AAAGCAAGCAAGAGTTCTATTACTAAACACAGCATGACTCAAAAAACTTA
					1551 GCAATTCTGAAGGAAAGTCCTTGGGGTCTTCTACGAGGAGTAGAATGTTG
					1601 AGAGTCAGCAGTAGCCTCATCATCACTAGATGGCATTTCTTCTGAGCAAA
					1651 ACAGGTTTTCCTCATTAAAGGCATTCCACCACTGCTCCCATTCATCAGTT
					1701 CCATAGGTTGGAATCTAAAATACACAAACAATTAGAATCAGTAGTTTAAC
					1751 ACATTATACACTTAAAAATTTTATATTTACCTTAGAGCTTTAAATCTCTG
					1801 TAGGTAGTTTG**TCCAATTATGTCACACCACAGAAGTAAGGTTCCTTCACA**
					1851 **AAGATCAAGTCCAAACCATCATTCTAAAGCAATCGAAGCAGTAGC**AATCA
			3	**2190–2274**	1901 ACCCACACAAGTGGATCTTTCCTGTATAATTTTCTATTTTCATGCTTCAT
					1951 CCTCAGTAAGCACAGCAAGCATATGCAGTTAGCAGACATTTTCTTTGCAC
					2001 ACTCAGGCCATTGTTTGCAGTACATTGCATCAACACCAGGATTTAAGGAA
					2051 GAAGCAAATACCTCAGTTGCATCCCAGAAGCCTCCAAAGTCAGGTTGATG
					2101 AGCATATTTTACTCCATCTTCCATTTTCTTGTACAGAGTATTCATCTTCA
					2151 TCTCCTCCTTTATCAGGATGAAACTCCTTGCATTTTTTT**AAATATGCCTT**
					2201 **TCTCATCAGAGGAATATTCCCCCAGGCACTCCTTTCAAGACCTAGAAGGT**
					2251 **CCATTAGCTGCAAAGATTCCTCTC**TGTTTAAAACTTTATCCAT
**E1A**	Adenoviral early region 1A gene	986			1 ATGAGACATATTATCTGCCACGGAGGTGTTATTACCGAAGAAAT**GGCCGC**
					51 **CAGTCTTTTGGACCAGCTGATCGAAGAGGTACTGGCTGATAATCTTCCAC**
					101 **CTCCTAGCCATTTT**TGAACCACCTACCCTTCACGAACTGTATGATTTAGAC
					151 GTGACGGCCCCCGAAGATCCCAACGAGGAGGCGGTTTCGCAGATTTTTCC
			1	**45–113**	201 CGACTCTGTAATGTTGGCGGTGCAGGAAGGGATTGACTTACTCACTTTTC
					251 CGCCGGCGCCCGGTTCTCCGGAGCCGCCTCACCTTTCCCGGCAGCCCGAG
					301 CAGCCGGAGCAGAGAGCCTTGGGTCCGGTTTCTATGCCAAACCTTGTACC
					351 GGAGGTGATCGATCTTACCTGCCACGAGGCTGGCTTTCCACCCAGTGACG
					401 ACGAGGATGAAGAGGGTGAGGAGTTTGTGTTAGATTATGTGGAGCACCCC
					451 GGGCACGGTTGCAGGTCTTGTCATTATCACCGGAGGAATACGGGGGACCC
					501 AGATATTATGTGTTCGCTTTGCTATATGAGGACCTGTGGCATGTTTGTCT
					551 ACAGTAAGTGAAAATTATGGGCAGTGGGTGATAGAGTGGTGGGTTTGGTG
					601 TGGTAATTTTTTTTTTAATTTTTACAGTTTTGTGGTTTAAAGAATTTTGT
					651 ATTGTGATTTTTTTAAAAGGTCCTGTGTCTGAACCTGAGCCTGAGCCCGA
					701 GCCAGAACCGGAGCCTGCAAGACCTACCCGCCGTCCTAAAATGGCGCCTG
			2	**874–940**	751 CTATCCTGAGACGCCCGACATCACCTGTGTCTAGAGAATGCAATAGTAGT
					801 ACGGATAGCTGTGACTCCGGTCCTTCTAACACACCTCCTGAGATACACCC
					851 GGTGGTCCCGCTGTGCCCCATTA**AACCAGTTGCCGTGAGAGTTGGTGGGC**
					901 **GTCGCCAGGCTGTGGAATGTATCGAGGACTTGCTTAACGA**GCCTGGGCAA
					951 CCTTTGGACTTGAGCTGTAAACGCCCCAGGCCATAA

Table S4: Platform-specific definitions of LOB, LOD and LOQ in digital PCR and countable PCR.

Assay Performance characteristic		Definition	Digital PCR	Countable PCR
**Limit of blank**	LOB	Highest expected signal from blank samples	NA	LOB = mean of blank reactions + 1.645 × SD of blank reactions
**Limit of detection**	LOD	Lowest signal level that can be distinguished from background	Minimum copy number concentration reliably detected, defined as all technical replicates being positive (≥3 positive droplets)	LOD = LOB + 1.645 × SD of reactions targeting 10 counts per reactions
**Limit of quantification**	LOQ	Lowest target level that can be quantified with predefined precision criteria	Mean copy number per reaction at the lowest target level where %CV < 20% across technical replicates	LOQ = Mean counts per reaction at the lowest target level where %CV ≤ 20% across technical replicates

Table S5: Quantitative bias between expected and measured copy numbers across the dilution series for each dPCR assay in different duplex combinations.

Target	dPCR assay combination	Expected copies/ rnx	Mean measured copies / rnx	%bias	Absolute % bias	Mean measured copies / rnx	%bias	Absolute % bias
				Assay 1 (or 2)			Assay 3	
Amp	1+3	80000	64882.0	-18.9	18.9	62358.7	-22.1	22.1
		10000	8972.0	-10.3	10.3	8108.5	-18.9	18.9
		2000	1789.4	-10.5	10.5	1606.7	-19.7	19.7
		1000	909.0	-9.1	9.1	815.7	-18.4	18.4
		100	89.6	-10.4	10.4	82.0	-18.0	18.0
		50	45.5	-9.0	9.0	40.1	-19.7	19.7
		20	20.1	0.7	0.7	18.4	-7.9	7.9
		10	9.5	-5.3	5.3	9.3	-7.2	7.2
	2+3	80000	66422.7	-17.0	17.0	62310.7	-22.1	22.1
		10000	8593.6	-14.1	14.1	8072.7	-19.3	19.3
		2000	1647.1	-17.6	17.6	1634.8	-18.3	18.3
		1000	881.5	-11.9	11.9	831.7	-16.8	16.8
		100	87.3	-12.7	12.7	83.1	-16.9	16.9
		50	44.5	-11.0	11.0	41.8	-16.4	16.4
		20	18.6	-7.1	7.1	17.2	-14.2	14.2
		10	11.1	10.9	10.9	10.5	4.7	4.7
Kan	1+3	80000	77253.3	-3.4	3.4	77309.4	-3.4	3.4
		10000	10190.6	1.9	1.9	10165.4	1.7	1.7
		2000	2104.7	5.2	5.2	2093.5	4.7	4.7
		1000	1017.8	1.8	1.8	1001.9	0.2	0.2
		100	110.0	10.0	10.0	106.2	6.2	6.2
		50	53.2	6.5	6.5	52.9	5.8	5.8
		20	22.4	11.9	11.9	21.5	7.7	7.7
		10	11.0	9.7	9.7	9.6	-3.6	3.6
	2+3	80000	73875.4	-7.7	7.7	77807.6	-2.7	2.7
		10000	9446.3	-5.5	5.5	10047.7	0.5	0.5
		2000	1906.5	-4.7	4.7	2027.5	1.4	1.4
		1000	932.2	-6.8	6.8	985.0	-1.5	1.5
		100	93.3	-6.7	6.7	95.3	-4.7	4.7
		50	49.6	-0.9	0.9	54.1	8.2	8.2
		20	21.7	8.4	8.4	22.2	10.9	10.9
		10	10.4	3.6	3.6	10.3	3.4	3.4
SV40T	1+3	80000	67672.4	-15.4	15.4	75993.5	-5.0	5.0
		10000	8503.9	-15.0	15.0	9532.9	-4.7	4.7
		2000	1747.7	-12.6	12.6	1963.4	-1.8	1.8
		1000	871.6	-12.8	12.8	980.1	-2.0	2.0
		100	96.8	-3.2	3.2	109.6	9.6	9.6
		50	44.3	-11.5	11.5	51.3	2.7	2.7
		20	20.0	0.0	0.0	21.9	9.5	9.5
		10	10.0	-0.4	0.4	12.3	23.2	23.2
	2+3	80000	70729.3	-11.6	11.6	78282.1	-2.1	2.1
		10000	9189.7	-8.1	8.1	9896.1	-1.0	1.0
		2000	1875.2	-6.2	6.2	2041.0	2.0	2.0
		1000	901.5	-9.9	9.9	973.6	-2.6	2.6
		100	101.9	1.9	1.9	111.0	11.0	11.0
		50	53.3	6.5	6.5	56.8	13.7	13.7
		20	19.0	-5.0	5.0	21.0	5.1	5.1
		10	11.6	16.2	16.2	11.8	17.7	17.7
E1A	1+2	80000	85173.5	6.5	6.5	89772.5	12.2	12.2
		10000	11091.2	10.9	10.9	11681.8	16.8	16.8
		2000	2251.2	12.6	12.6	2379.1	19.0	19.0
		1000	1097.3	9.7	9.7	1150.9	15.1	15.1
		100	121.4	21.4	21.4	127.2	27.2	27.2
		50	59.8	19.7	19.7	61.7	23.4	23.4
		20	23.6	18.0	18.0	19.4	-3.1	3.1
		10	14.3	43.4	43.4	14.7	46.8	46.8

Table S6: Restriction digest reactions details.

<table>
<tr><th>gBlock used</th><th>Reaction name</th><th>Restriction enzyme used</th><th>Units of the enzyme used in reaction</th><th>Buffer used</th><th>Incubation time (min) and temperature (°C)</th><th>Inactivation time and temperature (°C)</th><th>Expected number of fragments</th><th>Expected lengths of fragments (bp)</th></tr>
<tr><td rowspan="4">Amp</td><td>BaeGI</td><td>BaeGI</td><td>10</td><td rowspan="12">NEBuffer r3.1(New England Biolabs, NEB)</td><td rowspan="12">15
37</td><td rowspan="12">20
80</td><td>2</td><td>120
741</td></tr>
<tr><td>AseI</td><td>AseI</td><td>10</td><td>2</td><td>248
613</td></tr>
<tr><td>BaeGI+AseI</td><td>BaeGI
AseI</td><td>10
10</td><td>3</td><td>120
248
493</td></tr>
<tr><td>H_2O</td><td>/</td><td>/</td><td>1</td><td>861</td></tr>
<tr><td rowspan="4">Kan</td><td>AseI</td><td>AseI</td><td>10</td><td>2</td><td>183
633</td></tr>
<tr><td>Bpu10I</td><td>Bpu10I</td><td>5</td><td>2</td><td>364
452</td></tr>
<tr><td>AseI+Bpu10I</td><td>AseI
Bpu10I</td><td>10
5</td><td>3</td><td>181
183
452</td></tr>
<tr><td>H_2O</td><td>/</td><td>/</td><td>1</td><td>816</td></tr>
<tr><td rowspan="4">mSV40T</td><td>BaeGI</td><td>BaeGI</td><td>10</td><td>2</td><td>611
1682</td></tr>
<tr><td>BstXI</td><td>BstXI</td><td>10</td><td>2</td><td>383
1910</td></tr>
<tr><td>BaeGI+BstXI</td><td>BaeGI
BstXI</td><td>10
10</td><td>3</td><td>383
611
1299</td></tr>
<tr><td>H_2O</td><td>/</td><td>/</td><td>1</td><td>2293</td></tr>
<tr><td rowspan="2">E1A</td><td>SmaI</td><td>SmaI</td><td>20</td><td rowspan="2">rCutSmart Buffer (New England Biolabs, NEB)</td><td rowspan="2">15
37</td><td rowspan="2">20
65</td><td>2</td><td>450
536</td></tr>
<tr><td>H_2O</td><td>/</td><td>/</td><td>1</td><td>986</td></tr>
</table>

Table S7: Detailed capillary electrophoresis results showing the presence of unfragmented gBlocks or singly restricted gBlocks observed in the double-restriction reactions.

gBlock	Restriction enzyme used	Restriction location (A. between target 1 and 2, B. between targets 2 and 3).	% of gBlocks cleaved only at A	% of gBlocks cleaved only at B	% of unfragmented gBlocks
**Amp**	BaeGI+AseI	A&B	1.4	2.0	1.0
**Kan**	AseI+Bpu10I	A&B	17.6	0	2.1
**mSV40T**	BaeGI+BstXI	A&B	5.0	2.7	1.8

Table S8: Detailed performance characteristics of Countable PCR for ampicillin (Amp) and E1A assays' targets across a range of target counts per reaction.

	Amp						E1A			
	1.		2.		3.		1.		2.	
**Targeted counts per reaction**	Mean number of counts per reaction	%CV	Mean number of counts per reaction	%CV	Mean number of counts per reaction	%CV	Mean number of counts per reaction	%CV	Mean number of counts per reaction	%CV
0	2	80.8	2	127.3	4	50.8	2	94.4	3	78.5
10	11	35.5	8	33.7	10	37.6	12	42.9	12	38.5
20	23	27.9	19	27.2	18	38.0	21	43.1	24	33.6
50	52	20.0	42	20.6	43	21.2	51	22.8	55	17.1
60	60	21.3	50	25.2	48	26.8	57	18.4	56	22.6
75	78	18.6	67	18.5	71	46.9	65	17.4	63	29.1

Table S9: Summary of nanopore sequencing quality metrics.

Fraction	DNase pretreatment	Barcode	Number of reads	Mean read length (bp)	Median read length (bp)	Read length N50	Mean Q-score
**Heavy**	+	1	792495	2109.8	1290	4270	11.7
	-	2	1011054	1425.7	670	3557	11.9
**Full**	+	3	579458	2584.0	1888	4274	11.5
	-	4	842833	1917.0	1197.0	4251	11.6
**Intermediate**	+	5	648546	2571.1	1872.0	4268	11.6
	-	6	712012	2455.0	1841.0	4263	11.5
**Empty**	+	7	703046	2580.7	1876.0	4264	11.6
	-	8	675772	2537.9	1871.0	4264	11.5

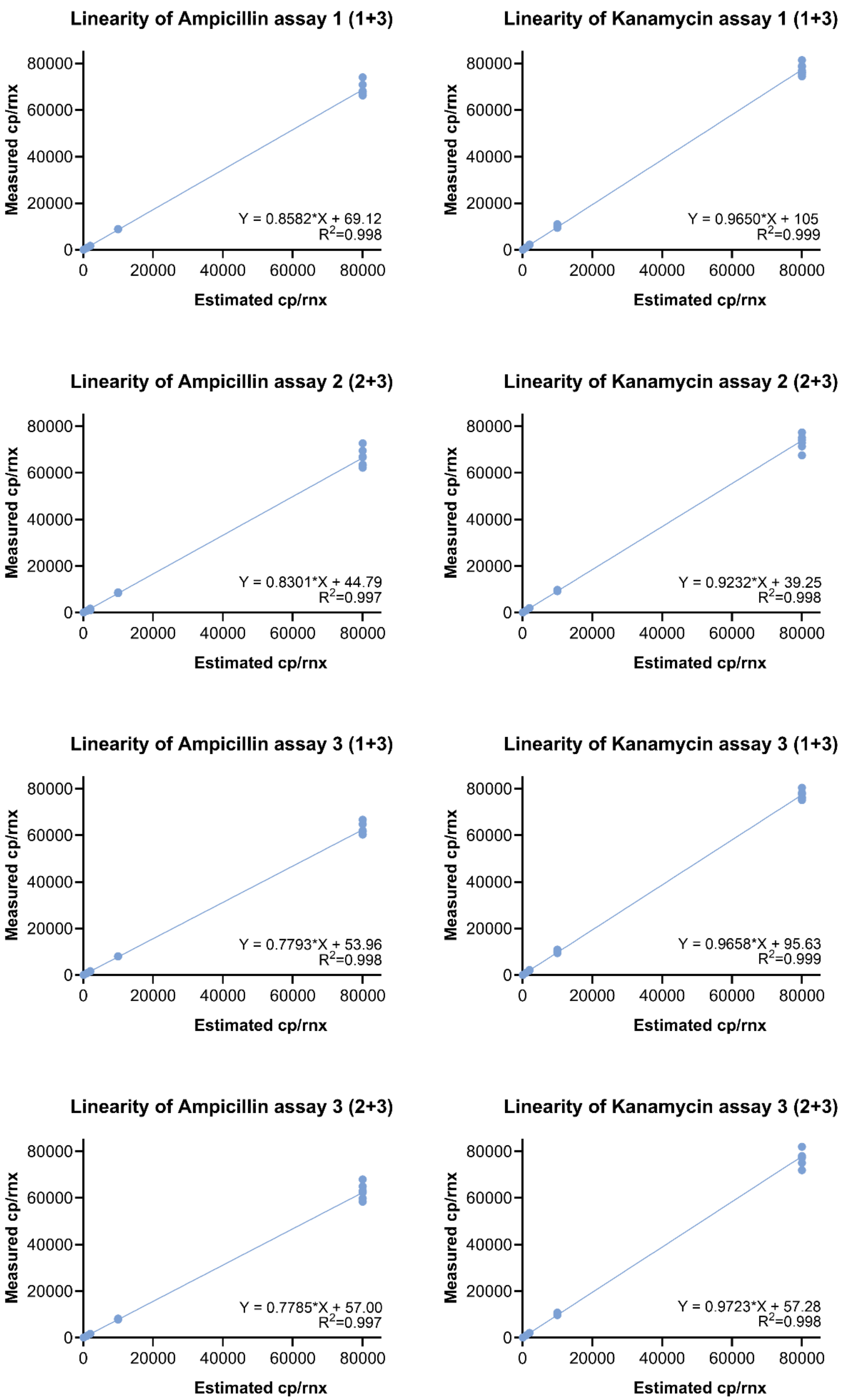

Figure S1: Determination of linearity for Ampicillin and Kanamycin digital PCR (dPCR) assays tested in double-duplex reaction setups. Assays 1 and 2 were used interchangeably in combination with assay 3. Linearity was determined for each assay in both duplex configurations.

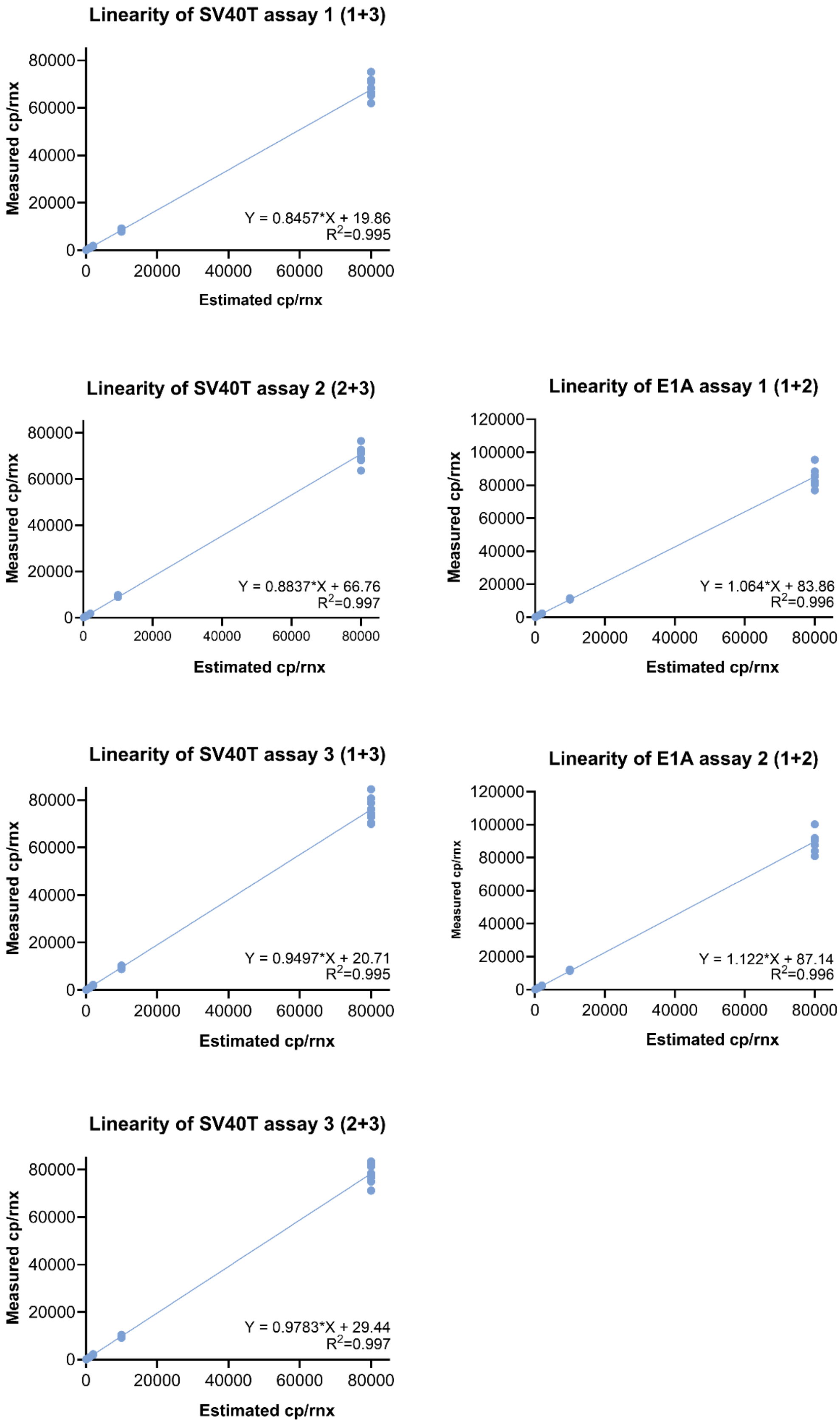

Figure S2: Determination of linearity for SV40T and E1A digital PCR (dPCR) assays tested in duplex reaction setups. Assays 1 and 2 were used interchangeably in combination with assay 3 in SV40T assay testing. Linearity was determined for each assay in both duplex configurations. E1A testing consisted of two targets only in one duplex setup.

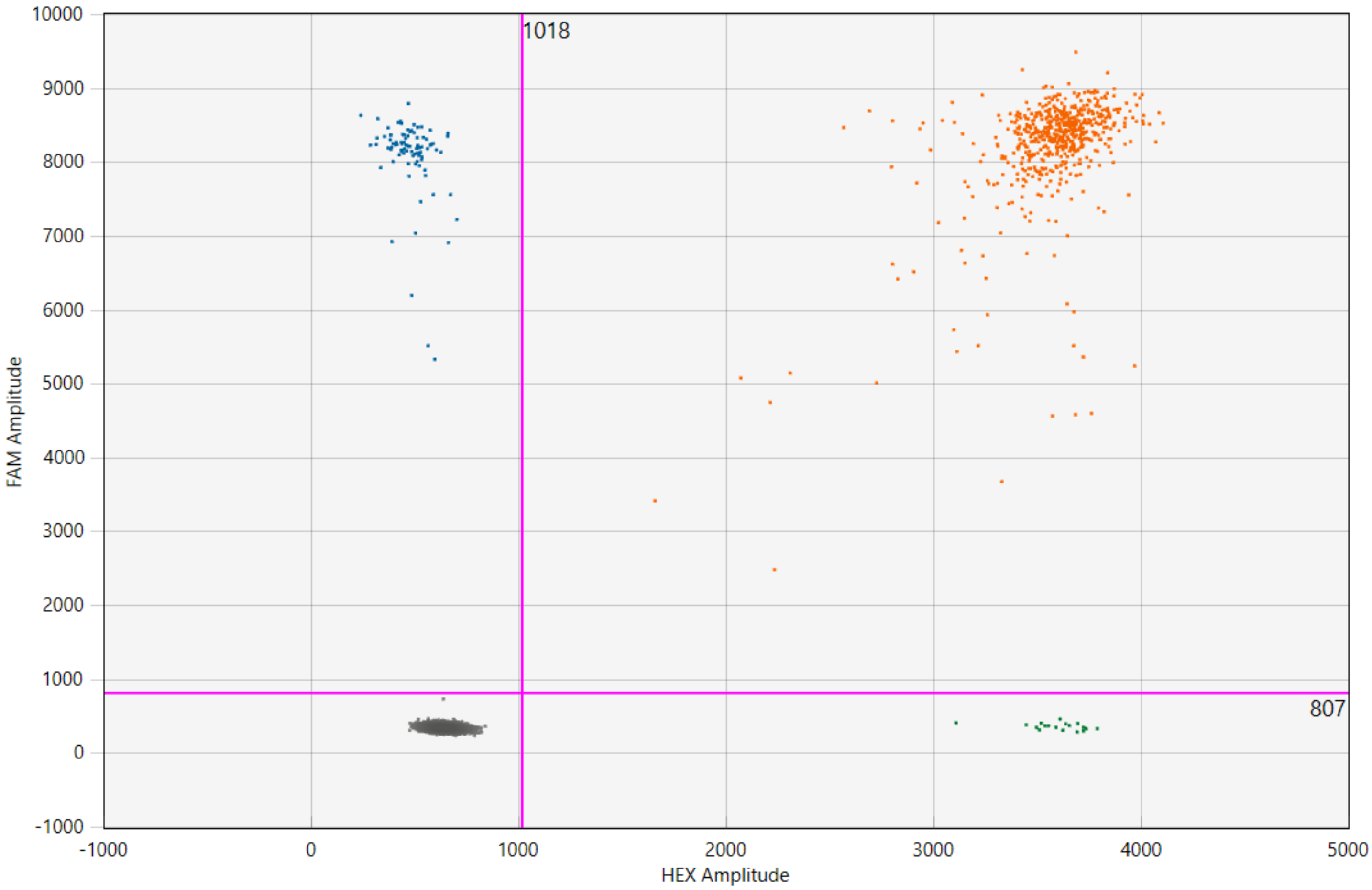

Figure S3: The 2D digital PCR plot of the unfragmented ampicillin gBlock using duplex assays. Representative two-dimensional (2D) digital PCR plot showing simultaneous detection in both fluorescence channels for the duplex assay. Assay 1 is labelled with FAM and assay 3 with HEX.

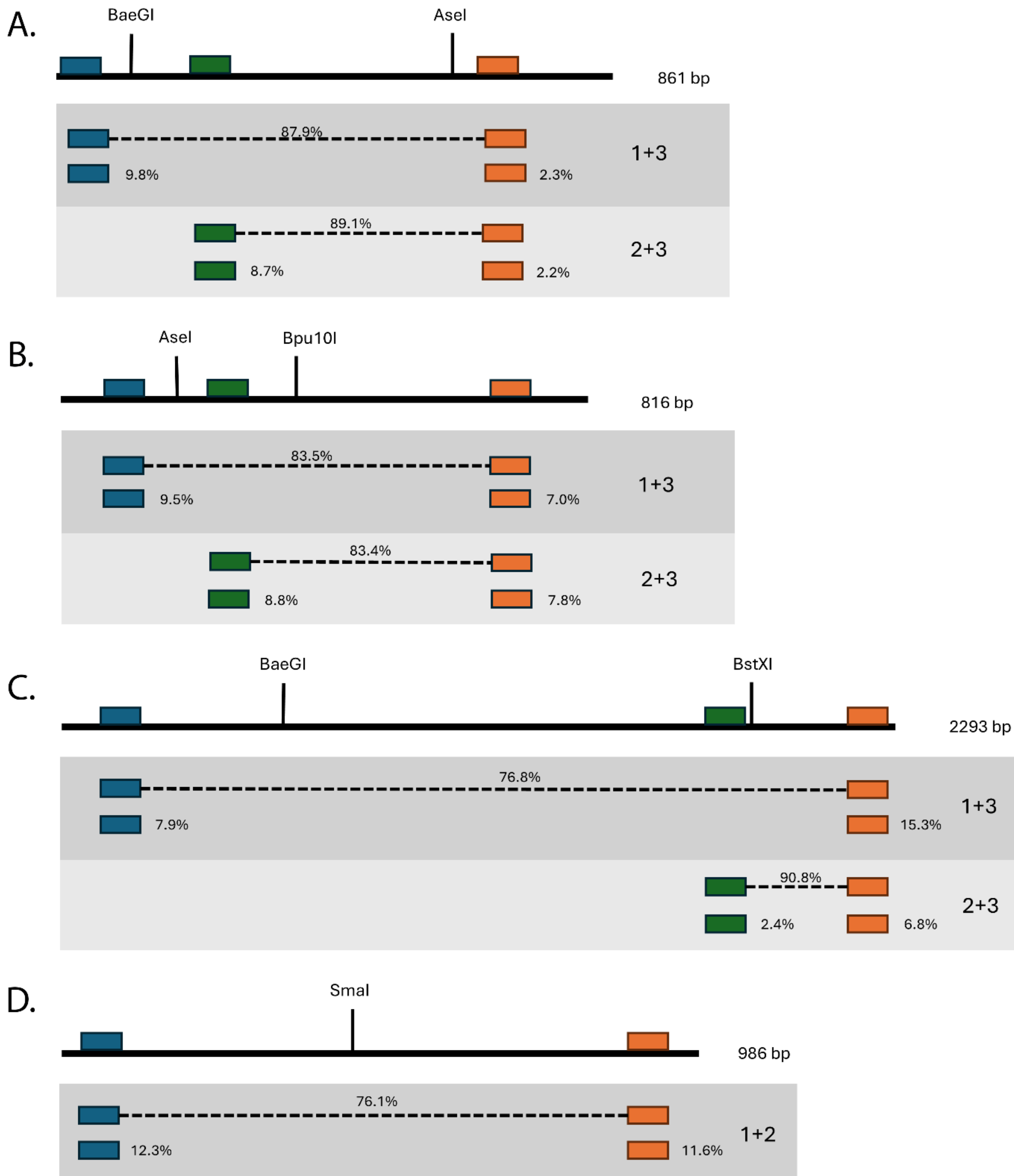

Figure S4: Detail dPCR results of unfragmented gBlocks testing. (A) Ampicillin gBlock. (B) Kanamycin gBlock. (C) mSV40T gBlock, (D) E1A gBlock. Minor deviations from 100% are attributable to rounding of individual values.

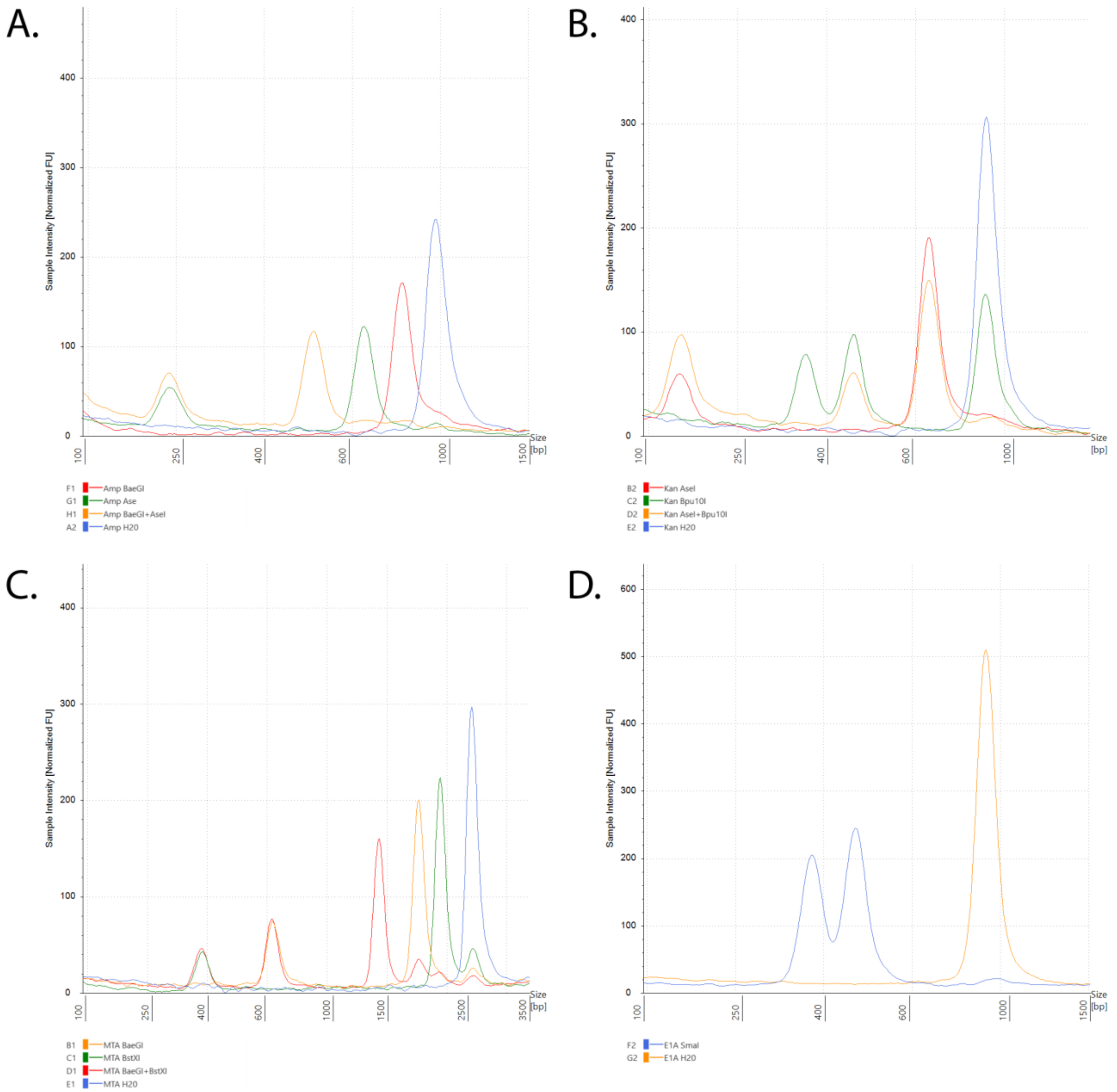

Figure S5: Restriction digest analysis of gBlocks by TapeStation. Electropherograms generated using TapeStation Analysis Software after restriction digestion of gBlocks with various enzymes (e.g., BaeGI, AseI, or a combination of BaeGI and AseI). Samples labeled "H_2O" represent undigested controls, where water was added instead of restriction enzyme. (A) Ampicillin gBlock, (B) Kanamycin gBlock, (C) mSV40T gBlock (named as MTA), and (D) E1A gBlock.

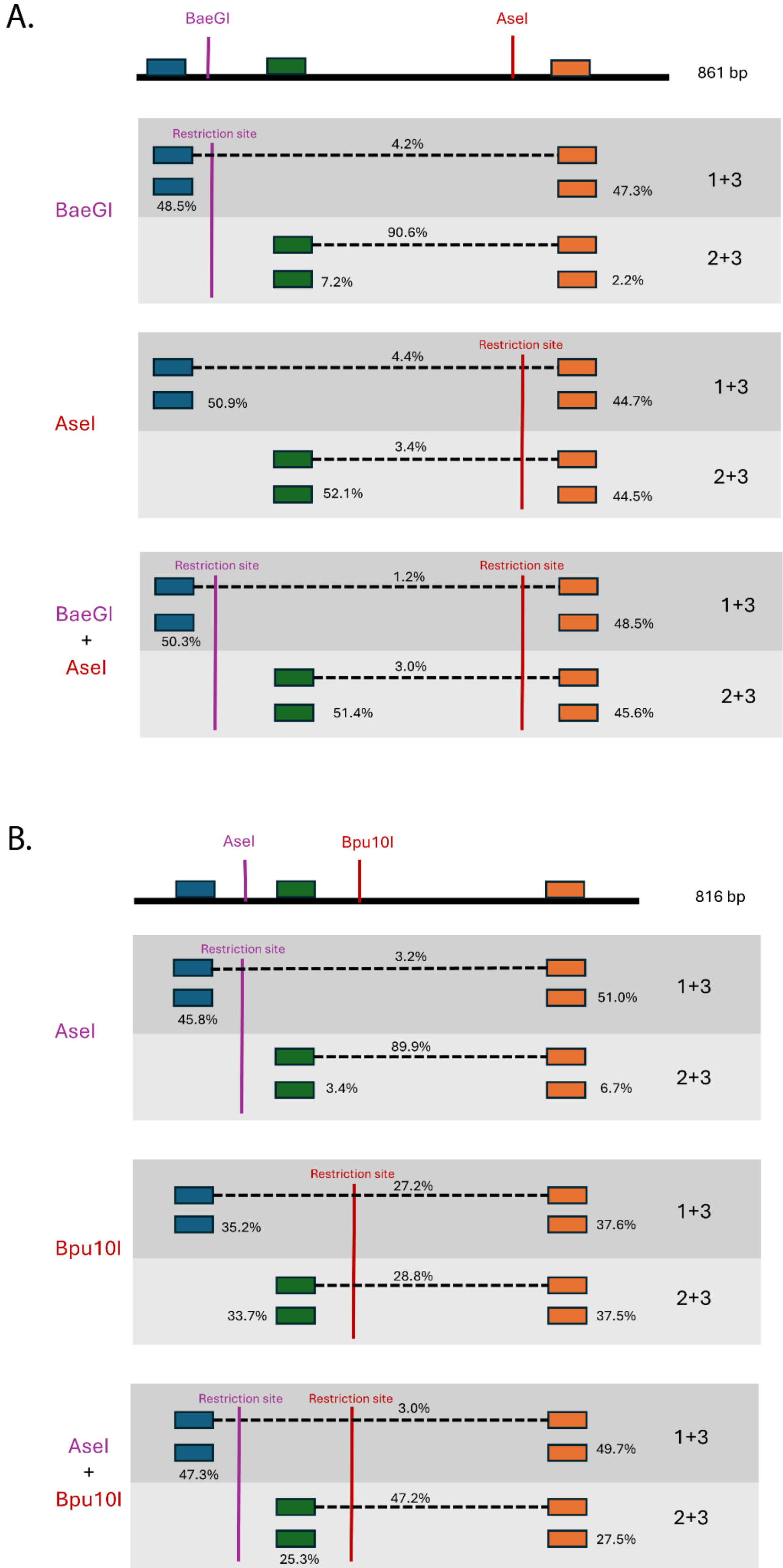

Figure S6: Detail dPCR results of restricted Ampicillin (A.) and Kanamycin (B) gBlocks testing. Ampicillin gBlock was digested with BaeGI or AseI, as well as with both enzymes simultaneously. Kanamycin gBlock was digested with AseI or Bpu10I, as well as with both enzymes. Minor deviations from 100% are attributable to rounding of individual values.

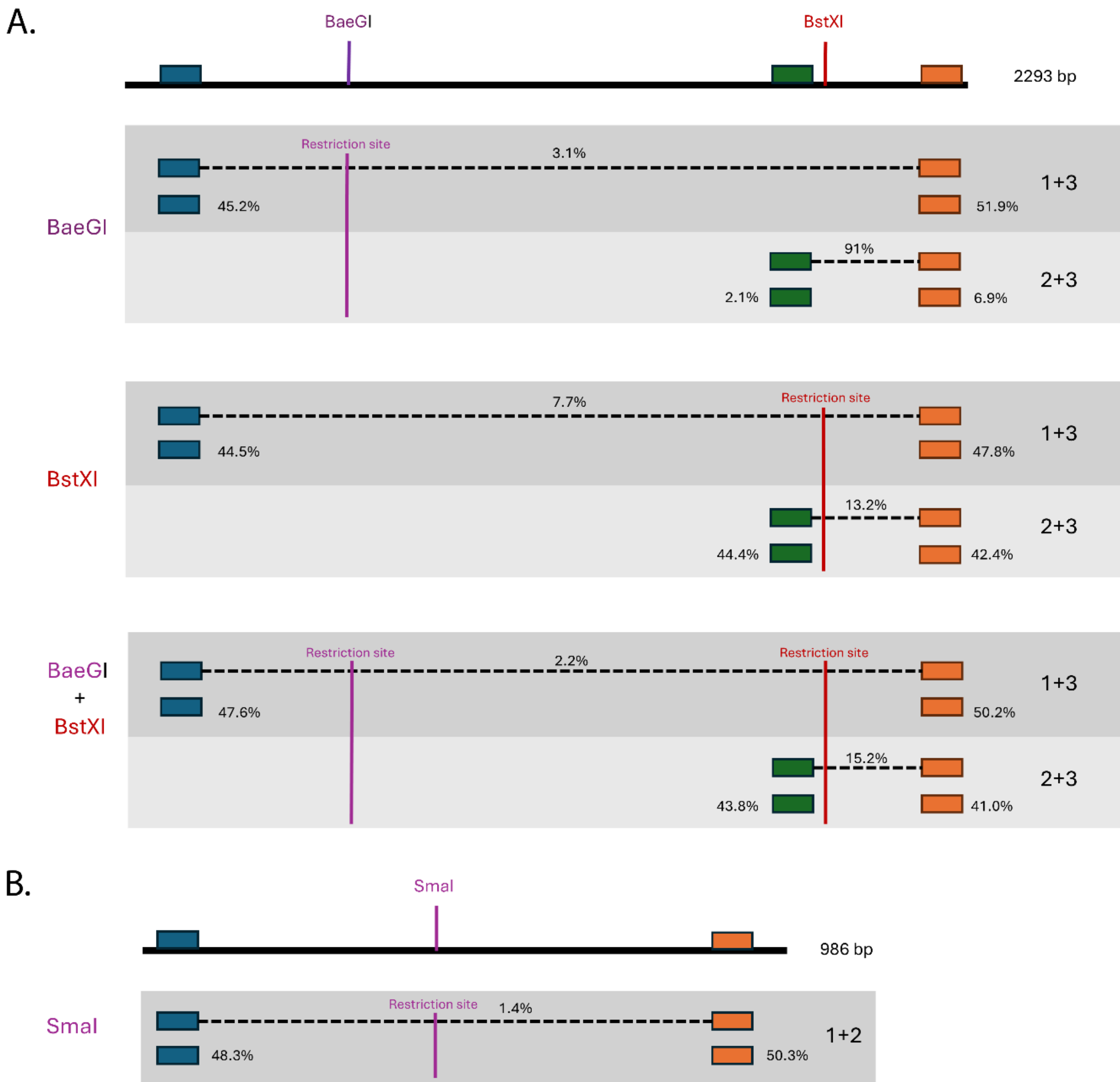

Figure S7: Detail dPCR results of restricted mSV40T (A) and E1A (B) gBlocks testing. mSV40T gBlock was digested with BaeGI or BstXI, as well as with both enzymes. E1A gBlock was digested with SmaI. Minor deviations from 100% are attributable to rounding of individual values.

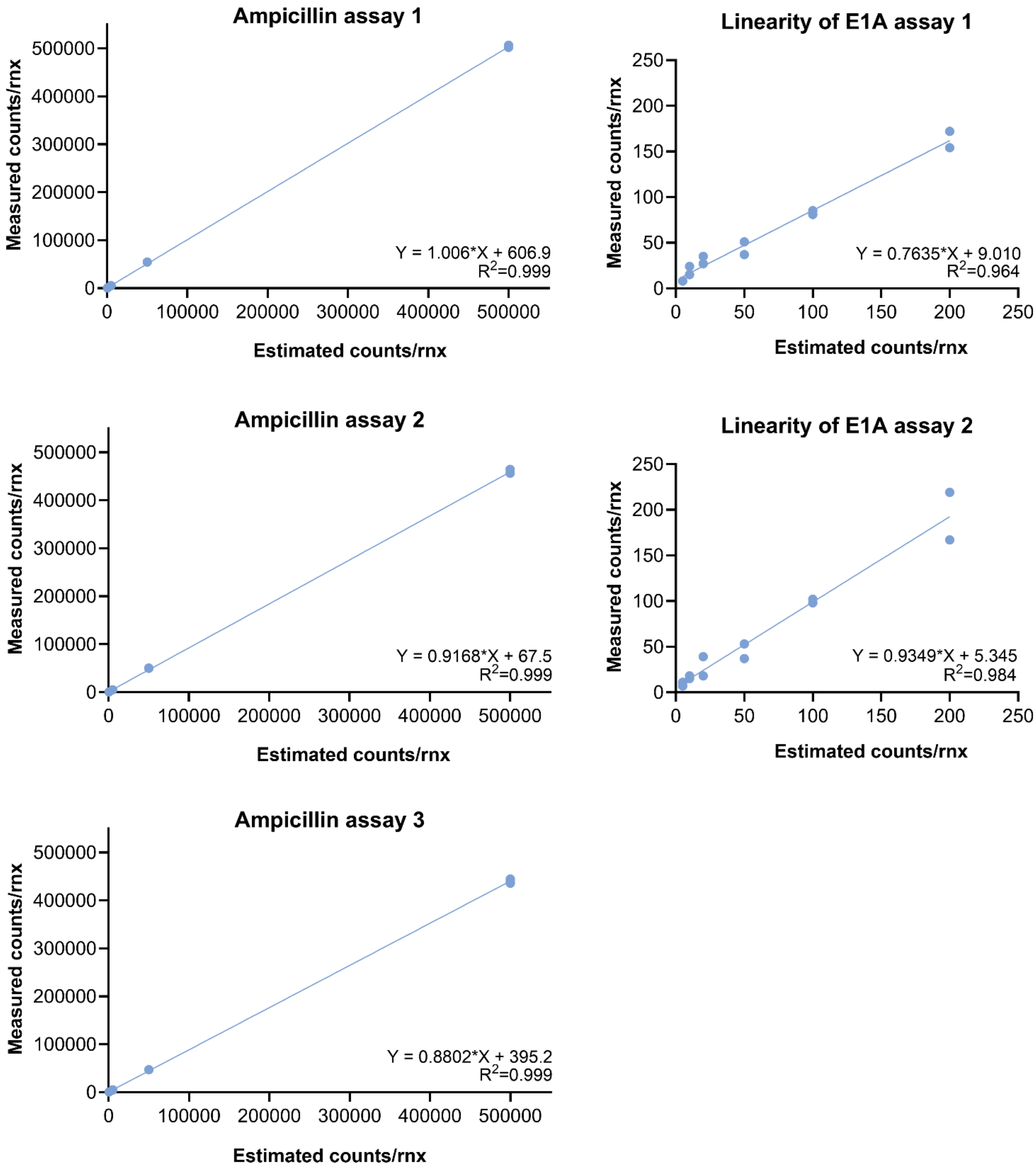

Figure S8: Linearity of countable PCR (cPCR) assays across dilution series, showing the relationship between expected and measured copies per reaction.

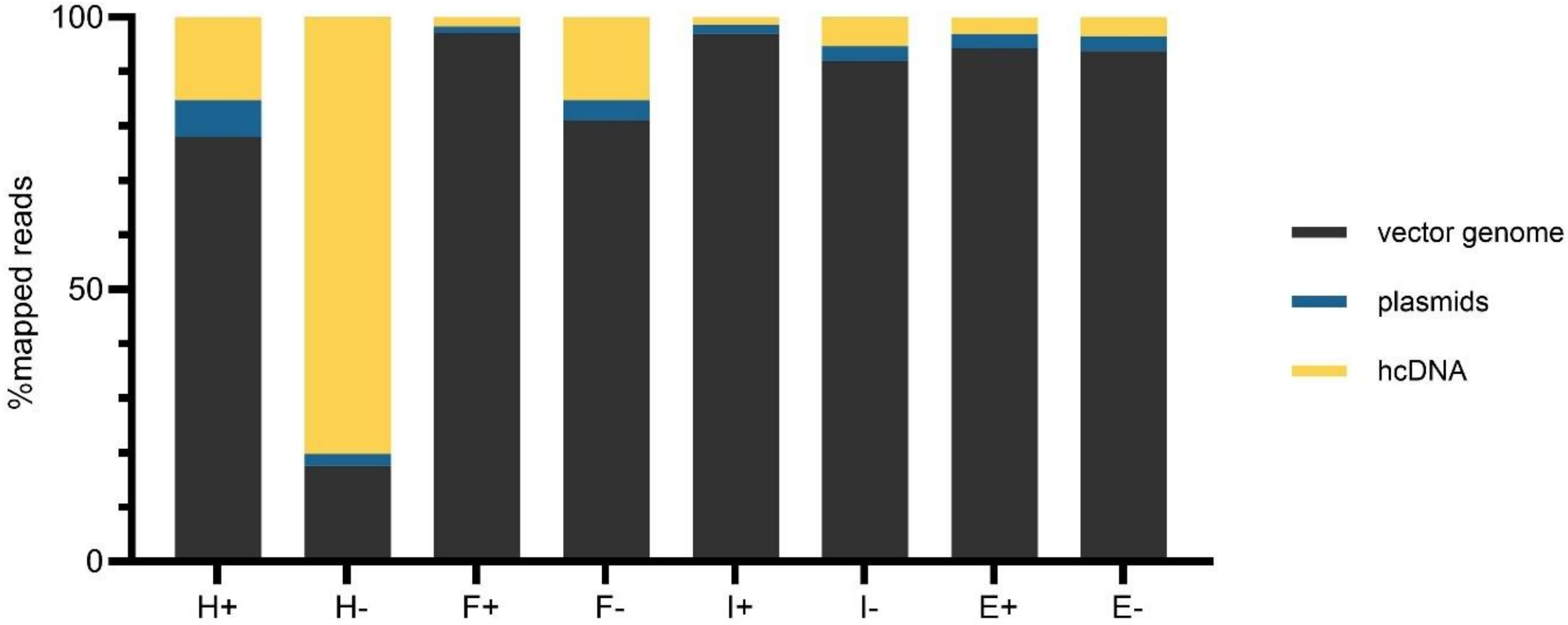

Figure S9: Nanopore read mapping results expressed as the % of total Nanopore sequencing reads mapping to either vector genome, plasmids or host cell DNA for each DNase I treated (+) and untreated sample (-). H = heavy, F = full, I = intermediate and E = empty fraction.

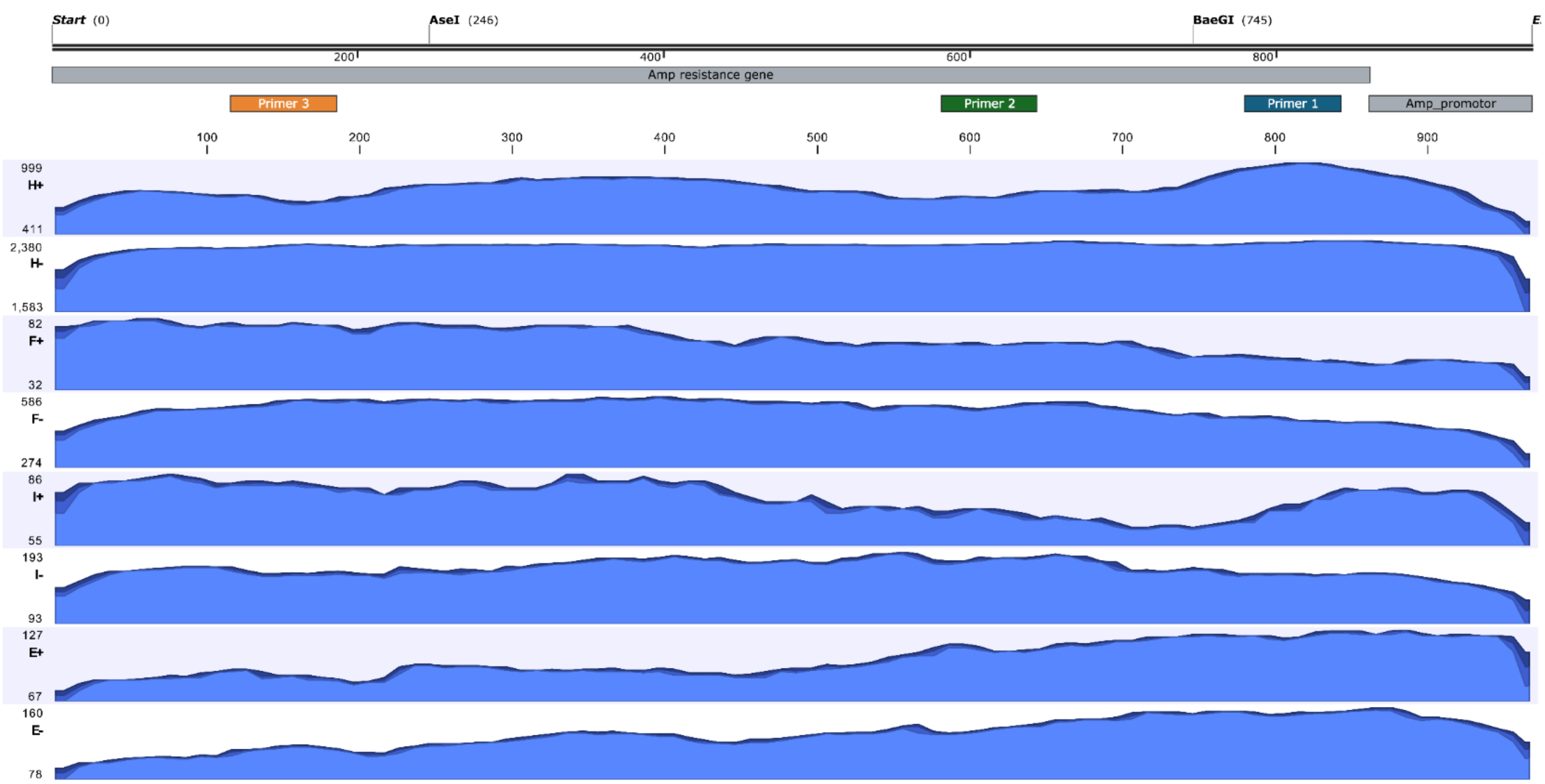

Figure S10: Coverage profiles of the ampicillin resistance gene in DNase I–treated (+) and untreated (–) fractions: heavy (H), full (F), intermediate (I), and empty (E). The top panel illustrates the reference genome containing the ampicillin resistance gene and its promoter, along with the genomic coordinates of the digital PCR assays developed in this study. Coverage tracks below represent read depth across the sequence. The x-axis indicates nucleotide position (bp), and the y-axis indicates read depth (number of reads mapped to each nucleotide position, linear scale). The values shown on the left of each track represent the minimum and maximum read depth observed within each sample.
